# Plasma Protein Biomarkers for the Prediction of CSF Amyloid and Tau and [^18^F]-Flutemetamol PET Scan Result

**DOI:** 10.3389/fnagi.2018.00409

**Published:** 2018-12-11

**Authors:** Sarah Westwood, Alison L. Baird, Abdul Hye, Nicholas J. Ashton, Alejo J. Nevado-Holgado, Sneha N. Anand, Benjamine Liu, Danielle Newby, Chantal Bazenet, Steven J. Kiddle, Malcolm Ward, Ben Newton, Keyur Desai, Cristina Tan Hehir, Michelle Zanette, Daniela Galimberti, Lucilla Parnetti, Alberto Lleó, Susan Baker, Vaibhav A. Narayan, Wiesje M. van der Flier, Philip Scheltens, Charlotte E. Teunissen, Pieter Jelle Visser, Simon Lovestone

**Affiliations:** ^1^Department of Psychiatry, University of Oxford, Oxford, United Kingdom; ^2^Maurice Wohl Clinical Neuroscience Institute, Institute of Psychiatry, Psychology and Neuroscience, King’s College London, London, United Kigndom; ^3^Biomedical Research Unit for Dementia, NIHR Biomedical Research Centre for Mental Health, South London and Maudsley NHS Foundation Trust, London, United Kingdom; ^4^Department of Psychiatry and Neurochemistry, Institute of Neuroscience and Physiology, Sahlgrenska Academy, University of Gothenburg, Mölndal, Sweden; ^5^Department of Biostatistics and Health Informatics, Institute of Psychiatry, Psychology & Neuroscience, King’s College London, London, United Kingdom; ^6^MRC Biostatistics Unit, Cambridge Biomedical Campus, Cambridge Institute of Public Health, University of Cambridge, Cambridge, United Kingdom; ^7^Proteomics Facility, Institute of Psychiatry, Psychology & Neuroscience, King’s College London, London, United Kingdom; ^8^GE Healthcare Life Sciences Core Imaging, London, United Kingdom; ^9^Biosciences, GE Global Research, Niskayuna, NY, United States; ^10^GE Healthcare Life Sciences Core Imaging, Marlborough, MA, United States; ^11^Neurodegenerative Diseases Unit, Centro Dino Ferrari, University of Milan, Milan, Italy; ^12^Fondazione IRCCS Ca’ Granda Ospedale Maggiore Policlinico, Milan, Italy; ^13^Center for Memory Disorders and Laboratory of Clinical Neurochemistry, Neurology Clinic, University of Perugia, Perugia, Italy; ^14^Department of Neurology, Hospital de la Santa Creu i Sant Pau, Biomedical Research Institute Sant Pau, Universitat Autònoma de Barcelona, Barcelona, Spain; ^15^Janssen Neuroscience Research & Development, Titusville, NJ, United States; ^16^Department of Neurology, Alzheimer Centre, Amsterdam Neuroscience, VU University Medical Center, Amsterdam, Netherlands; ^17^Department of Epidemiology and Biostatistics, VU University Medical Center, Amsterdam, Netherlands; ^18^Department of Clinical Chemistry, Neurochemistry Lab and Biobank, Amsterdam Neuroscience, VU University Medical Center, Amsterdam, Netherlands; ^19^Department of Psychiatry and Neuropsychology, School for Mental Health and Neuroscience, Maastricht University, Maastricht, Netherlands

**Keywords:** Alzheimer’s disease, amyloid, tau, biomarkers, proteomics, plasma, blood, ficolin-2

## Abstract

**Background:** Blood biomarkers may aid in recruitment to clinical trials of Alzheimer’s disease (AD) modifying therapeutics by triaging potential trials participants for amyloid positron emission tomography (PET) or cerebrospinal fluid (CSF) Aβ and tau tests.

**Objective:** To discover a plasma proteomic signature associated with CSF and PET measures of AD pathology.

**Methods:** Liquid chromatography-tandem mass spectrometry (LC-MS/MS) based proteomics were performed in plasma from participants with subjective cognitive decline (SCD), mild cognitive impairment (MCI), and AD, recruited to the Amsterdam Dementia Cohort, stratified by CSF Tau/Aβ_42_ (*n* = 50). Technical replication and independent validation were performed by immunoassay in plasma from SCD, MCI, and AD participants recruited to the Amsterdam Dementia Cohort with CSF measures (*n* = 100), MCI participants enrolled in the GE067-005 study with [^18^F]-Flutemetamol PET amyloid measures (*n* = 173), and AD, MCI and cognitively healthy participants from the EMIF 500 study with CSF Aβ_42_ measurements (*n* = 494).

**Results:** 25 discovery proteins were nominally associated with CSF Tau/Aβ_42_ (*P* < 0.05) with associations of ficolin-2 (FCN2), apolipoprotein C-IV and fibrinogen β chain confirmed by immunoassay (*P* < 0.05). In the GE067-005 cohort, FCN2 was nominally associated with PET amyloid (*P* < 0.05) replicating the association with CSF Tau/Aβ_42_. There were nominally significant associations of complement component 3 with PET amyloid, and apolipoprotein(a), apolipoprotein A-I, ceruloplasmin, and PPY with MCI conversion to AD (all *P* < 0.05). In the EMIF 500 cohort FCN2 was trending toward a significant relationship with CSF Aβ_42_ (*P* ≈ 0.05), while both A1AT and clusterin were nominally significantly associated with CSF Aβ_42_ (both *P* < 0.05).

**Conclusion:** Associations of plasma proteins with multiple measures of AD pathology and progression are demonstrated. To our knowledge this is the first study to report an association of FCN2 with AD pathology. Further testing of the proteins in larger independent cohorts will be important.

## Introduction

The accumulation of amyloid-beta (Aβ) plaques followed by the deposition of hyper-phosphorylated tau protein in neurofibrillary tangles, central to Alzheimer’s disease (AD) neuropathology, is thought to develop around 20 to 30 years in advance of clinical symptom onset (Jansen et al., 2015). Given the long prodromal phase of the disease, a biomarker of these early neuropathological changes would be beneficial in participant selection and cohort enrichment for clinical trials of disease modifying therapies targeting AD neuropathology.

To date the best characterized and most frequently used biomarkers relating to amyloid and tau pathology are PET imaging measures of brain amyloid deposition and cerebrospinal fluid (CSF) measures of Aβ, total tau (tTau) and phospho-tau (pTau) (Kang et al., 2013; Palmqvist et al., 2015). However, PET scans can be expensive and access to scanners and radioligands remains limited, whilst extracting CSF is relatively invasive and can therefore be problematic to obtain, particularly if repeated measures are required. Blood based biomarkers have therefore been investigated as a less invasive and potentially cost-effective option for early detection and monitoring of AD pathology.

Many studies have investigated blood-based proteomic biomarkers to distinguish Alzheimer’s disease cases from cognitively healthy elderly controls as reviewed (Thambisetty and Lovestone, 2010; Fu et al., 2014). However, to date there has been a relatively low rate of replication of these biomarkers across the field. This may be in part due to issues surrounding a study design that compares AD to cognitively healthy elderly control subjects. Given that AD neuropathology precedes clinical presentation of the disease by a number of years, some cognitively healthy elderly subjects may in fact be harboring silent AD neuropathology. This reduces the ability to find biomarkers specifically relating to AD using this design, as some control subjects will instead be preclinical cases. To overcome this issue biomarkers specific to the underlying disease have been sought including candidates relating to rate of cognitive decline, progression from mild cognitive impairment (MCI) to AD or disease pathology (MRI measures of brain atrophy, measures of brain amyloid) as we have reviewed earlier (Baird et al., 2015).

In such studies predicated not on disease category but on ‘endophenotypes’ of disease, our group has previously identified protein markers in blood relating to brain atrophy, disease severity and progression and to accumulation of cerebral amyloid as measured using PET imaging (Thambisetty et al., 2010; Kiddle et al., 2012; Hye et al., 2014; Sattlecker et al., 2014; Ashton et al., 2015; Voyle et al., 2015; Westwood et al., 2016). This has included the identification of a panel of 10 proteins, which coupled with *APOE* genotype, could predict MCI conversion to AD with 87% accuracy (Hye et al., 2014). Moreover, we have previously identified a number of proteins associated with PET amyloid in both an AD based cohort (Ashton et al., 2015) and in cognitively healthy elderly (Westwood et al., 2016). Notably, one protein; fibrinogen gamma chain (FGG), when combined with age was able to predict neocortical amyloid burden with 59% sensitivity and 78% specificity (Ashton et al., 2015).

However to date we are not aware of any studies that have been designed to discover blood protein biomarkers relating to CSF measures of AD pathology including both measures of Aβ_42_ and of tau. Therefore, in the present study we first sought to discover and then to perform a technical replication of candidate biomarkers correlating with and predicting CSF Tau/Aβ_42_ pathology in samples collected in the Amsterdam Dementia Cohort at VU University medical center, using an untargeted mass spectrometry proteomic approach. Secondly, we aimed to validate these candidates by relating their levels to disease pathology and progression in two independent cohorts. Firstly, we utilized plasma from people with MCI who were also assessed with brain amyloid PET using [^18^F]Flutemetamol [GE067-005 study (Wolk et al., 2018)], measuring in these samples both the proteins identified in discovery and replication phase and protein markers of endophenotypes identified previously in the studies referenced above. Secondly, we utilized plasma from AD, MCI and cognitively healthy control individuals sourced through the European Medical Information Framework (EMIF) platform^1^ who also had a CSF Aβ_42_ measure, here we focused on measuring protein markers of AD pathology identified in the discovery and replication phase and also from our previous studies. Our overall aim was identification, replication and validation of blood markers indicative of brain pathology that might be used to reduce screening failure when seeking to recruit people with pathology to clinical trials.

## Materials and Methods

### Study Participants, Assessments, Blood Collection and Processing

#### Amsterdam Dementia Cohort

Biomarker discovery proteomics were performed on plasma samples from participants with AD, MCI or with mild subjective cognitive decline (SCD) visiting the Alzheimer Center Amsterdam as previously described (van der Flier et al., 2014; van der Flier and Scheltens, 2018). In brief, the diagnosis of probable AD was made according to common clinical and research criteria (McKhann et al., 1984, 2011), for MCI the Petersen’s criteria was used (Petersen et al., 1999) and SCD was assigned to patients who presented with cognitive complaints but did not meet the criteria for dementia, MCI or any neurological or psychiatric conditions affecting cognition (van der Flier et al., 2014). Venous blood samples were processed for plasma and stored according to international consensus standard operating procedures (Teunissen et al., 2009).

Plasma samples were selected on the basis of CSF Aβ_42_ and tau measures and, using an extreme phenotype approach, were stratified as very low CSF pathology (low CSF tau/high CSF Aβ_42_, *n* = 25) to very high CSF pathology (high CSF tau/low CSF Aβ_42_, *n* = 25) (Table 1). Calculation of the CSF pathology score was carried out using the discrimination line: *x* = (373 + 0.82[tau])/[Aβ_42_] (Mulder et al., 2010).

**Table 1:** Demographics of subjects from the Amsterdam Dementia Cohort.

Variable	Subjects included in LC-MS/MS discovery			Subjects included in ELISA technical replication		
	Low CSF pathology	High CSF pathology	*P*-value	Low CSF pathology	High CSF pathology	*P*-value
*N*	25	25	/	50	50	/
CSF pathology = (373+0.82[tau])/[Aβ42]	0.56 ± 0.05	3.63 ± 0.49	<0.001^∗^	0.54 ± 0.05	3.45 ± 0.82	<0.001^∗^
Age (years)	65.84 ± 3.80	65.01 ± 3.81	0.648	63.74 ± 4.51	64.21 ± 4.13	0.549
Female gender N (%)	10 (40)	11 (44)	0.777	21 (42)	27 (54)	0.232
Clinical diagnosis						
SCD N (%)	16 (64)	2 (8)	/	41 (82)	5 (10)	/
MCI N (%)	8 (32)	7 (28)	/	8 (16)	7 (14)	/
AD N (%)	1 (4)	16 (64)	/	1 (2)	38 (76)	/
*APOE* genotype 𝜀4+ N (%)	6 (24)	18 (72)	<0.01^∗^	16 (32)	30 (60)	<0.01^∗^
MMSE	28 ± 2	25 ± 2	<0.001^∗^	28 ± 2	22 ± 6	<0.001^∗^

*CSF, cerebrospinal fluid; SCD, subjective cognitive decline; MCI, mild cognitive impairment; AD, Alzheimer’s disease; *APOE*, apolipoprotein E; MMSE, mini-mental state examination. Mean ± SD. ^∗^Statistically significant.*

Replication studies using immunocapture techniques of the biomarkers identified in the discovery phase were carried out in the plasma samples included in the discovery phase and in additional plasma samples from a further 50 subjects visiting the Alzheimer Center Amsterdam, also stratified by CSF pathology score (Mulder et al., 2010). In total 50 plasma samples per group were included in the replication phase (Table 1). Data from clinical assessments were available for all subjects including mini mental state examination (MMSE) and Apolipoprotein E (*APOE*) genotype data (van der Flier et al., 2014).

#### GE067-005 Study

Plasma samples from MCI participants enrolled in the GE067-005 study^2^ (Wolk et al., 2018) were included in the independent validation study (*n* = 173, Table 2). This included amnestic MCI subjects who converted to probable AD within a 3-year time frame (MCI Converter, *n* = 52) and subjects who remained MCI over this time period (MCI non-converter, *n* = 121). Participants were assessed clinically every 6 months until conversion, dropout or completion of the 3-year follow-up. [^18^F]Flutemetamol PET amyloid imaging data were available for all subjects, who were categorized as having either an “abnormal” PET amyloid scan (positive for the presence of amyloid, *n* = 68) or a “normal” PET amyloid scan (negative for the presence of amyloid, *n* = 105). Visual image interpretation of the PET scan was performed by five independent trained readers who were blinded to the participants’ clinical history and diagnosis. Scan interpretation was based on the majority classification from these five independent readers. The details of image interpretation are published elsewhere (Wolk et al., 2018). General clinical and demographic data were also available for all subjects, including *APOE* genotype, body mass index (BMI), prevalence of diabetes and years of education. Whole blood was collected in EDTA tubes and processed for plasma (Supplementary Methods Section 1).

**Table 2:** Demographics of the subjects from the GE067-005 study.

Variable	GE067-005 subjects grouped by PET amyloid			GE067-005 subjects grouped by MCI conversion		
	Low [18F] PET amyloid	High [18F] PET amyloid	*P-*value	MCI non-converters	MCI converters	*P*-value
*N*	105	68	/	121	52	/
High PET amyloid N (%)	/	/	/	36 (30)	32 (62)	<0.001^∗^
PET uptake value	1.25 ± 0.14	2.05 ± 0.36	<0.001^∗^	1.46 ± 0.41	1.81 ± 0.50	<0.001^∗^
MCI converter N (%)	20 (19)	32 (47)	<0.001^∗^	/	/	/
Age (years)	69.13 ± 8.73	73.37 ± 7.64	<0.01^∗^	69.36 ± 8.39	74.15 ± 8.04	<0.01^∗^
Female gender N (%)	50 (48)	37 (54)	0.384	59 (49)	28 (54)	0.541
BMI	27.42 ± 4.73	25.63 ± 3.47	<0.05^∗^	26.86 ± 4.63	26.39 ± 3.64	0.916
Education (years)	13.28 ± 3.54	13.72 ± 4.11	0.706	13.21 ± 3.63	14.00 ± 4.07	0.135
Diabetes N (%)	9 (9)	5 (7)	0.775	10 (8)	4 (8)	0.900
*APOE* genotype 𝜀4+ N (%)	23 (22)	41 (60)	<0.001^∗^	37 (31)	27 (52)	<0.01^∗^

*PET, [^*18*^F]-flutemetamol positron emission tomography; MCI, mild cognitive impairment; BMI, body mass index; *APOE*, apolipoprotein E. Mean ± SD. ^∗^Statistically significant.*

#### EMIF 500 Study

The EMIF (see text footnote 1) is a precompetitive, public-private Innovative Medicines Initiative funded platform facilitating access to cohort studies and real world observational data from across Europe. We used EMIF to identify three cohorts with samples suitable for validation studies. Plasma samples from AD, MCI and cognitively healthy participants (CTL), for whom a measure of CSF Aβ_42_, tTau and pTau was available, were included in this independent validation study (*n* = 494, Table 3). Participants were recruited by three separate centers; Clinica Neurologica, Universita di Perugia (*n* = 252), Hospital Sant Pau, Barcelona (*n* = 154) and the Alzheimer Unit of Fondazione Ca Granda, IRCCS Ospedale Maggiore Policlinico, Milan (*n* = 88). Diagnoses were made according to standard criteria (McKhann et al., 1984, 2011; Petersen et al., 1999) and general clinical and demographic information were available for all subjects (including MMSE and *APOE* genotype data). CSF Aβ_42_, tTau and pTau cut-off values for assigning high and low categories were provided by each individual cohort; Perugia, Aβ_42_ = 800 pg/mL, tTau = 300 pg/mL, pTau = 60 pg/mL (Majbour et al., 2017); Barcelona; Aβ_42_ = 550 pg/mL, tTau = 350 pg/mL, pTau = 61 pg/mL (Alcolea et al., 2015); and Milan, Aβ_42_ = 600 pg/mL, tTau = 450 pg/mL, pTau = 61 pg/mL (Galimberti et al., 2014). CSF Aβ_42_, tTau and pTau values across cohorts were combined into continuous variables using z-scoring. For each CSF measure, *Z*-scores were calculated independently for each cohort before being combined into one variable.

**Table 3:** Demographics of the subjects from the EMIF 500 study.

Variable	EMIF 500 subjects grouped by CSF Aβ42		
	Low CSF Aβ42	High CSF Aβ42	*P*-value
*N*	198	294	/
Age (years)	66.21 ± 9.73	69.76 ± 8.81	<0.001^∗^
Female gender N (%)	112 (57)	167 (57)	0.959
AD N (%)	9 (5.6)	152 (94.4)	/
MCI N (%)	116 (49.6)	118 (50.4)	/
CTL N (%)	73 (75.3)	24 (24.7)	/
*APOE* genotype 𝜀4+ N (%)	43 (22)	147 (50)	<0.001^∗^

*MCI, mild cognitive impairment; *APOE*, apolipoprotein E. Mean ± SD. ^∗^Statistically significant.*

### Discovery Phase: Gel LC-MS/Mass Spectrometry Based Proteomics

Discovery proteomics was carried out by gel LC-MS/Mass Spectrometry (LC-MS/MS) coupled with tandem mass tagging (TMT). The data acquisition and preprocessing pipelines are described in detail elsewhere (Ashton et al., 2015). In brief, the proteomics workflow consisted of plasma samples labeled in a TMT6plex configuration with TMT126 – TMT130 (Thermo Fisher Scientific), and the study reference with TMT131. The tagged samples within each TMT6plex were pooled and then separated by one-dimensional gel electrophoresis. Ten equal fractions were excised from each gel and the gel pieces were destained, tryptically digested and peptides extracted. LC-MS/MS acquisition was performed using the Orbitrap Velos Pro instrumentation (Thermo Fisher Scientific) coupled to a Proxeon EASY-nLC II system (Thermo Fisher Scientific). The LC-MS/MS raw Excalibur data files (Thermo Fisher Scientific) were processed by Proteome discover (Thermo Fisher Scientific, version 1.3) using Mascot^3^ (version 2.3) to determine peptide identifications. Processing of the Mascot output data files was performed in R, and included median ratio normalization, calculation of peptide ratios and subsequent protein scores using median and mean roll-up methods of peptide ratios as described (Ashton et al., 2015). Where the same protein was observed by electrophoresis in multiple different molecular weight regions of the one-dimensional gel, these observations were treated as separate protein molecular weight isoforms in subsequent analysis. Analysis of each protein molecular weight isoform was performed on the data produced from both the mean and median protein roll up methods and age, sex, *APOE* 𝜀4 allele presence and sample storage duration were included as covariates in regression models.

### Replication Phase: Immunocapture Assay Based Proteomics

Technical replication was performed on proteins in the same 50 samples that underwent LC-MS/MS based proteomics with an additional 50 samples selected from the Amsterdam Dementia Cohort (Table 1). The criteria for protein selection for replication included; (1) nominal statistical significance, (2) quantification by ≥2 peptides, and (3) detection by 1D gel electrophoresis in the molecular weight range of the native protein. Eight proteins were selected for replication; ficolin-2 (FCN2), apolipoprotein C-IV (ApoC-IV), c4-binding protein alpha chain (C4BPA), fibrinogen β chain (FGB), Ig gamma-3 chain C region (IGHG3), apolipoprotein A-I (ApoA-I), serotransferrin and apolipoprotein A-IV (ApoA-IV). These proteins included novel candidates and proteins that had previously been identified in AD based biomarker studies (Yu et al., 2003; Liu et al., 2006; Thambisetty et al., 2010; Ijsselstijn et al., 2011; Hu et al., 2012; Song et al., 2012; Hye et al., 2014; Ashton et al., 2015; Westwood et al., 2016).

Proteins were measured by commercially available single-analyte enzyme-linked immunosorbent assays (ELISAs) according to the manufacturer’s instructions (Supplementary Table 1). ELISA absorbance at 450 nm was detected using a microplate reader (PHERAstar FS, BMG, LABTECH). The background corrected (570 nm) absorbance data were exported into Sigma Plot (Systat Software; version 12.5) for estimation of protein concentrations using a 5-parameter logistic fit. Intra-assay and inter-assay variability was assessed by calculation of the percentage coefficient of variation (% CV). Average intra-assay CVs were calculated using the duplicate measures for each sample and average inter-assay CV was calculated using the measurement of a control sample, which was analyzed on each plate. All protein concentration values were log_10_ transformed prior to statistical analysis and age, sex, *APOE* 𝜀4 allele presence, plate batch and sample storage duration were included as covariates in regression models.

### Independent Validation Phase

Proteins were selected from the replication phase in the Amsterdam Dementia Cohort, based upon nominal statistical significance, for further extension studies using the GE067-005 cohort. In addition, other proteins previously identified as markers related to AD pathology and progression, including candidate markers of MCI conversion to AD (Hye et al., 2014), rates of cognitive decline and disease severity and brain atrophy (Hye et al., 2014; Sattlecker et al., 2014) and [^11^C] Pittsburgh compound B (PiB) PET amyloid (Kiddle et al., 2012; Ashton et al., 2015; Voyle et al., 2015; Westwood et al., 2016) were also selected (Supplementary Table 2). In total 37 targets (including alpha-2-macroglobulin measured by two different assays) were measured in the GE067-005 study cohort (Supplementary Table 2). Of these, twenty-six proteins were measured by multiplex bead assays across 7 MagPlex MAP panels using the Luminex 200 instrument (Supplementary Table 1). Median fluorescent intensity (MFI) was measured using xPONENT 3.1 (Luminex Corporation). Eleven proteins were quantified by commercially available ELISAs as described earlier (Supplementary Table 1). Intra-assay and inter-assay variability was calculated as described earlier. All protein concentration values were log_10_ transformed prior to statistical analysis in order to achieve a normal distribution and the following covariates were included in regression models: age, sex, *APOE* 𝜀4 allele presence, BMI, diabetes, center, batch variation and sample storage duration.

Proteins were also selected from our earlier discovery studies for independent validation in the EMIF 500 study cohort. In total 21 proteins were measured by multiplex bead assays using the Luminex 200 instrument and by commercially available ELISAs as described earlier (Supplementary Table 1). As plasma proteins were measured in singular, the data were assessed for outliers on a protein-by-protein basis. Extreme outliers were defined as values falling outside three times the inter-quartile range of all samples measured and were removed from the dataset prior to subsequent statistical analysis. The following covariates were included in regression models: age, sex, *APOE* 𝜀4 allele presence, center, and batch variation.

### Statistical Procedures

#### Univariate Statistical Analysis

All statistical analyses were performed in R (version 1.3.3). For both discovery and replication phase studies, proteomic data were analyzed using the Mann Whitney *U*-test and logistic regression to compare dichotomized high and low CSF pathology groups. The association between proteomic data and continuous measures of CSF Tau/Aβ_42_ were assessed by Spearman rank correlation and linear regression. Validation phase studies were analyzed by both linear and logistic regression to assess the relationship between the proteomic data and pathology endophenotypes when accounting for covariates. Benjamini-Hochberg *q* values were calculated as a multiple testing correction for all analyses.

#### Classification Analysis for the Prediction of Amyloid Status

A generalized linear regression model (GLM) was used to adjust the data for covariates. Machine learning (i.e., classification) was performed in R on the GLM adjusted data. The minimal protein set with optimal AUC characteristics for prediction of amyloid status were assessed by Support Vector Machines combined with LASSO, as a variable selection method, and performance was assessed using 100 repeats of 10-fold cross validation.

#### Pathway Analysis: Using LC-MS/MS Proteomic Data

Differential regulation of pathways associated with CSF Tau/Aβ_42_ pathology were identified through gene enrichment analysis on the results of the LC-MS/MS analysis. For this analysis only protein molecular weight isoforms detected in 80% or more of the TMT6plexs and for which gene IDs were available were included. The *p*-values derived from the univariate analysis of the protein molecular weight isoforms were used to estimate a single *p*-value per protein by applying Fisher’s method as described here. For each protein the sum of logarithms of the *p*-values of all the molecular weight isoforms were calculated. The chi-squared distribution was then used to derive the protein *p*-value from this sum of logarithms.

We next expanded the analysis to include proteins that directly interact with the proteins detected in 80% or more of the TMT6plexs. Firstly, the *p*-values were log transformed and then the proteins known to interact with these proteins were identified by STRING (Szklarczyk et al., 2015). Only the most stringent protein-protein interactions (that have direct experimental evidence) were considered, with a confidence level >0.4. For each STRING protein, the average of the normalized *p*-values of the proteins that directly interact with them was then calculated.

Enrichment analysis on the expanded list proteins was performed using the Kolmogorov Smirnov test (KS test) with gene-lists corresponding to pathways [Reactome; (Joshi-Tope et al., 2005)], diseases [DisGeNet; (Pinero et al., 2015)], and GWAS studies [GWAS catalog; (Welter et al., 2014)]. The *p*-values were corrected with a permutation test of 50,000 iterations.

## Results

**FIGURE 1:**
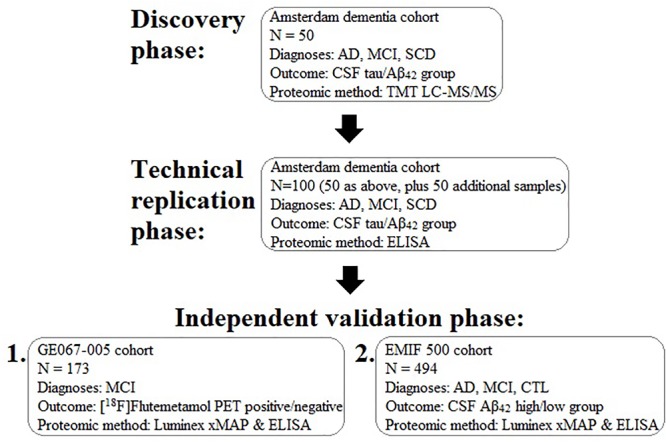
Schematic diagram illustrating the experimental work flow of the present study for the discovery, replication, and validation of plasma proteins associated with brain pathology. AD, Alzheimer’s disease; MCI, mild cognitive impairment; SCD, subjective cognitive decline; CSF, cerebrospinal fluid; TMT, tandem mass tagging; LC-MS/MS, liquid chromatography tandem mass spectrometry; Aβ, amyloid-beta.

In order to identify a biomarker that might predict brain amyloid pathology we performed a proteomic study in three phases in three independent sample collections. First, we used mass-spectrometry based discovery in the Amsterdam Dementia Cohort including a technical replication phase using immunocapture. Then we used immunocapture to replicate these and previous findings in a cohort derived from a clinical trial of a radiotracer for detection of brain amyloid (GE067-005 study) and finally an independent validation phase study, again using immunocapture methods in an independent set of samples collated from three separate cohorts sourced using the EMIF. The study workflow is illustrated in Figure 1.

### Discovery Phase: Gel LC-MS/MS Based Proteomics in the Amsterdam Dementia Cohort

Using multiplexed mass spectrometry, we identified 804 protein MW isoforms, constituting 249 unique known protein groups from the combined MS/MS runs (Supplementary Table 3). Of these proteins, 253 protein MW isoforms, consisting of 85 unique protein groups were identified in 80% or more of TMT6plex’s (Supplementary Table 4). 42 protein MW isoforms corresponding to 25 unique proteins passed 1 or more statistical tests assessing the relationship with CSF pathology group or continuous measures of CSF Tau/Aβ_42_ (all nominal *P* < 0.05, Supplementary Table 5).

### Replication Phase: Immunocapture Assay Based Proteomics in the Amsterdam Dementia Cohort

In order to further assess these discovery phase observations, we performed a combined technical and clinico-biological replication study for eight proteins. Intra-assay CV was <13% for all assays, and batch variation was included as a covariate in regression analysis to control for any inter-assay differences.

**FIGURE 2:**
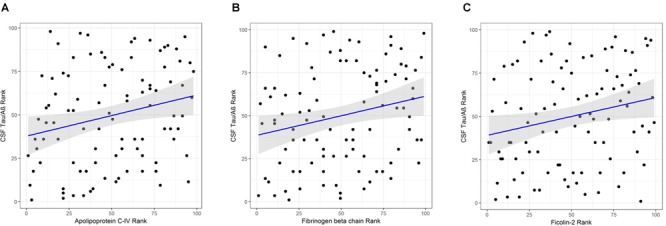
**(A)** Correlation of APOC-IV rank with CSF Tau/Aβ rank. **(B)** Correlation of FGB rank with CSF Tau/Aβ rank. **(C)** Correlation of ficolin-2 rank with CSF Tau/Aβ rank.

Apolipoprotein C-IV was nominally significantly increased in the high CSF Tau/Aβ_42_ pathology group compared to the low CSF Tau/Aβ_42_ pathology group by logistic regression (β = 0.665, *P* < 0.05; Table 4). ApoC-IV, FGB and FCN2 were also positively correlated with CSF Tau/Aβ_42_ by Spearman rank correlation (*r* = 0.237, *P* < 0.05, Figure 2A; and *r* = 0.228, *P* < 0.05, Figure 2B; *r* = 0.214, *P* < 0.05, Figure 2C, respectively, Table 4), replicating the results observed in the LC-MS/MS discovery data. Correlations of ApoC-IV and FGB with the separate CSF measures of Aβ_42_ and pTau were also observed by Spearman’s rank correlation and linear regression (Supplementary Table 6). ApoC-IV was negatively associated with CSF Aβ_42_ by both linear regression and spearman rank correlation (β = -101.867, *P* < 0.01 and *r* = -0.300, *P* < 0.01, respectively) and FGB was positively correlated with CSF pTau by spearman rank correlation (*r* = 0.227, *P* < 0.05).

**Table 4:** ELISA data: associations of the proteins with CSF tau/Aβ pathology.

UniProt ID	Protein name	Logistic regression			Mann-Whitney U			Linear regression			Spearman’s rank correlation			Number of tests with a *P*-value < 0.05
		β	*P*-value	*q*-value	Median difference	*P*-value	*q*-value	β	*P*-value	*q-*value	Rho	*P*-value	*q*-value	
P55056	Apolipoprotein C-IV	0.665	0.040^∗^	0.320	0.415	0.099	0.264	0.281	0.075	0.511	0.237	0.019^∗^	0.088	2
P02675	Fibrinogen beta chain	0.141	0.593	0.677	0.541	0.074	0.264	0.141	0.437	0.511	0.228	0.023^∗^	0.088	1
Q15485	Ficolin-2	0.343	0.275	0.616	0.171	0.097	0.264	0.232	0.201	0.511	0.214	0.033^∗^	0.088	1
P04003	C4b-binding protein alpha chain	–0.169	0.524	0.677	0.046	0.572	0.763	–0.061	0.741	0.741	0.165	0.104	0.207	0
P06727	Apolipoprotein A-IV	0.212	0.423	0.676	0.149	0.423	0.678	0.149	0.389	0.511	0.117	0.248	0.388	0
P01860	Ig gamma-3 chain C region	0.107	0.718	0.718	0.041	0.754	0.823	0.137	0.447	0.511	0.107	0.291	0.388	0
P02787	Serotransferrin	–0.539	0.133	0.531	–0.401	0.146	0.293	–0.185	0.348	0.511	–0.098	0.366	0.418	0
P02647	Apolipoprotein A-I	0.299	0.308	0.616	0.081	0.823	0.823	0.252	0.178	0.511	0.076	0.457	0.457	0

*ELISA, enzyme-linked immunosorbent assay; UniProt, universal protein resource; Aβ, amyloid-beta. CSF tau/Aβ was calculated using the equation: *x* = *373* + *0.82* (tau)/Aβ42. For linear and logistic regression the covariates: age, gender, *APOE* 𝜀4 allele presence, sample storage duration and ELISA batch were included. ^∗^Statistically significant *P* < 0.05*.

### Pathway Analysis: LC-MS/MS Proteomics in the Amsterdam Dementia Cohort

In order to explore the potential biological significance of these findings, we then performed a pathway analysis. The total summed *p*-values were calculated for 77 proteins from 233 protein MW isoform *p*-values for inclusion in gene enrichment analysis. Using STRING this list was expanded to include proteins for which there is direct experimental evidence of an interaction, giving a total of 769 proteins. When comparing this protein set to the Reactome database, three pathways were significant after FDR correction for multiple comparisons; HDL-mediated lipid transport (*q* = 0.010), lipoprotein metabolism (*q* = 0.035), and lipid digestion, mobilization, and transport (*q* = 0.035) (Table 5). Comparison to the DisGeNet database revealed three diseases were significant post FDR correction; hypercholesterolemia, familial (*q* = 0.034), brain diseases (*q* = 0.034), and metabolic bone disorder (*q* = 0.034) (Table 5).

**Table 5:** Pathway analysis of the proteins associated with CSF Tau/Aβ, comparing gene lists corresponding to pathways (Reactome), diseases (DisGeNet), and GWAS studies (GWAS catalog).

Database #	Pathway name	*P*-value	
		FDR corrected (*q*-value)	Uncorrected
***Reactome***			
R-HSA-194223	HDL-mediated lipid transport	0.0104	0.0001
R-HSA-174824	Lipoprotein metabolism	0.0347	0.0009
R-HSA-73923	Lipid digestion, mobilization, and transport	0.0347	0.0010
R-HSA-174800	Chylomicron-mediated lipid transport	0.1404	0.0054
R-HSA-196741	Cobalamin (Cbl, vitamin B12) transport and metabolism	0.2149	0.0124
R-HSA-174577	Activation of C3 and C5	0.2149	0.0189
R-HSA-196791	Vitamin D (calciferol) metabolism	0.2149	0.0196
R-HSA-975634	Retinoid metabolism and transport	0.2149	0.0198
R-HSA-381426	Regulation of Insulin-like Growth Factor transport and uptake by Insulin-like Growth Factor Binding Proteins	0.2149	0.0206
R-HSA-556833	Metabolism of lipids and lipoproteins	0.2149	0.0207

**UMLS #**	**Disease name**	***P*-value**	
		**FDR corrected (*q*-value)**	**Uncorrected**

***DisGeNet***			
C0020445	Hypercholesterolemia, familial	0.0344	0.0005
C0006111	Brain diseases	0.0344	0.0009
C0005944	Metabolic bone disorder	0.0344	0.0013
C0020476	Hyperlipoproteinemias	0.0569	0.0032
C0020615	Hypoglycemia	0.0569	0.0036
C0497327	Dementia	0.0569	0.0046
C0017661	IGA glomerulonephritis	0.0569	0.0052
C0030567	Parkinson disease	0.0569	0.0058
C0035126	Reperfusion injury	0.0569	0.0062
C0005612	Birth weight	0.0647	0.0085

**EFO/GO#**	**Trial name**	***P*-value**	
		**FDR corrected (*q*-value)**	**Uncorrected**

***GWAS Catalog***			
EFO 0004571	Butyrylcholinesterase measurement	0.0721	0.0047
EFO 0000319	Cardiovascular disease	0.0721	0.0090
EFO 712	Stroke	0.0721	0.0092
EFO 3892	Pulmonary function measurement	0.0721	0.0122
EFO 0004746	Lipoprotein-associated phospholipase a(2) measurement	0.0721	0.0215
EFO 0004723	Coronary artery calcification	0.0721	0.0219
EFO 0004214	Abdominal aortic aneurysm	0.0721	0.0235
EFO 0004624	Prostate specific antigen measurement	0.0721	0.0241
GO 0042493	Response to drug	0.0721	0.0295
EFO 0004461	Iron biomarker measurement	0.0721	0.0374

### Independent Validation Phase: Immunocapture Assay Based Proteomics in the GE067-005 Study Cohort

Using immunocapture, we then performed a validation phase study in an independent cohort using a different end point measure of AD pathology ([^18^F]-Flutemetamol PET amyloid). From 37 proteins measured, three proteins were excluded from analysis due to technical failure of the assays (Soluble receptor for advanced glycation end products, Complement C4-B and IGHG3). Intra-assay CV was <12% for all other assays, and batch variation was included as a covariate in regression analysis to control for any inter-assay differences.

#### Univariate Analysis

Increased FCN2 levels in the [^18^F]-flutemetamol PET positive group were observed by logistic regression (β = 0.580, *P* < 0.05, Supplementary Table 7), replicating the relationship with CSF Tau/Aβ_42_ observed in the Amsterdam Dementia Cohort. A nominally significant association of Complement C4 with [^18^F]-flutemetamol PET amyloid was also observed by both logistic regression (β = 0.750, *P* < 0.05, Supplementary Table 7) and linear regression (β = 0.079, *P* < 0.05, Supplementary Table 7). Apolipoprotein(a) [Apo(a)], ApoA-I, Ceruloplasmin and Pancreatic prohormone were all nominally associated with MCI conversion to AD by logistic regression (β = -0.476, *P* < 0.05; β = 0.631, *P* < 0.05; β = -0.526, *P* < 0.05; β = -0.456, *P* < 0.05, respectively, Supplementary Table 8).

#### Classification Analysis for the Prediction of Amyloid Status in the GE067-005 Cohort ([^18^F]-Flutemetamol PET Group)

**FIGURE 3:**
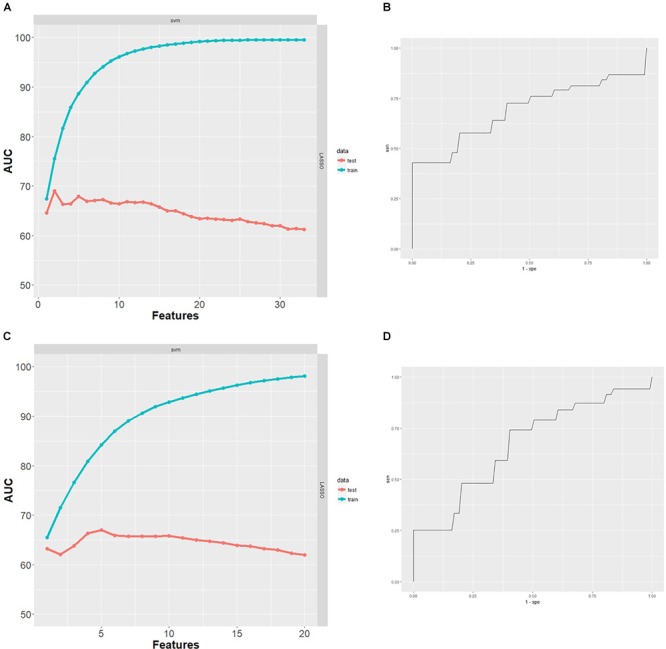
**(A)** Performance of SVM classifiers built using *n* = 1–33 proteins, ranked by LASSO and with 100 repeats of 10-fold cross validation. Two proteins were the minimal protein set with optimal AUC for classifying [^18^F]-Flutemetamol PET positivity in the GE067-005 cohort. **(B)** Receiver operating characteristics (ROC) curve obtained for the minimal two protein classifier (Aβ40 and ApoC4) for prediction of [^18^F]-Flutemetamol PET positivity in the GE067-005 cohort. **(C)** Performance of SVM classifiers built using *n* = 1–20 proteins, ranked by LASSO and with 100 repeats of 10-fold cross validation. Five proteins were the minimal protein set with optimal AUC for classifying CSF Aβ_42_ positivity in the EMIF 500 cohort. **(D)** ROC curve obtained for the minimal five protein classifier (A1AT, HAGP, Ig Kappa chain C region, PEDF, and RANTES) for prediction of CSF Aβ_42_ positivity in the EMIF 500 cohort.

After excluding subjects with missing data, the classification analysis was performed on 78 GE067-005 subjects. These subjects were split between amyloid-positive and amyloid-negative groups as follows: negative [^18^F]-Flutemetamol PET, *n* = 44; positive [^18^F]-Flutemetamol PET, *n* = 34 as measured by visual inspection according to the approved methods for image interpretation. The minimal protein set with optimal AUC characteristics for prediction of amyloid group was assessed by SVM combined with LASSO and performance was assessed using 100 repeats of 10-fold cross validation. Two proteins (Aβ40 and ApoC4) formed the minimal protein panel for classifying amyloid positivity and achieved modest accuracy (AUC = 0.69 (Figure 3), PPV = 0.52, NPV = 0.51, sensitivity = 0.57, and specificity = 0.44).

### Independent Validation Phase in the EMIF 500 Study Cohort

#### Univariate Statistical Analysis of Plasma Proteins in Relation to CSF Aβ_42_

In order to explore the relationship between these proteins and disease we obtained samples from three cohorts sourced through EMIF and including participants with MCI and AD as well as normal controls. We first considered the relationship between plasma proteins and CSF Aβ_42_ pathology in all AD, MCI and CTL subjects combined. In the high Aβ_42_ pathology group a reduction in A1AT (β = -0.248, *P* < 0.05, Supplementary Table 9) and an increase in clusterin (β = 0.278, *P* < 0.05, Supplementary Table 9) were observed by logistic regression. There was also a trend toward increased FCN2 in the high Aβ_42_ pathology group (β = 0.216, *P* = 0.055, Supplementary Table 9). A1AT was also nominally significantly associated with CSF Aβ_42_ pathology by linear regression (β = 31.690, *P* < 0.05, Supplementary Table 9).

The relationship between the 21 plasma proteins and CSF Aβ_42_ were then examined within each of the separate diagnostic groups (*n* = 162 AD, *n* = 235 MCI, *n* = 97 CTL). In the AD group, only RANTES was associated with CSF Aβ_42_, as shown by both logistic regression (β = -1.192, *P* < 0.01, Supplementary Table 10) and linear regression (β = 35.759, *P* < 0.05, Supplementary Table 10). In MCI there was an association of ICAM1 with CSF Aβ_42_ by linear regression (β = 66.327, *P* < 0.01, Supplementary Table 11). In the CTL group, a significant decrease in CFHR-1 and FGG in association with high CSF Aβ_42_ by logistic regression (β = -1.435, *P* < 0.05 and β = -1.003, *P* < 0.05, respectively, Supplementary Table 12) was observed, whilst A1AT was nominally significantly associated with CSF Aβ_42_ by both logistic and linear regression (β = -1.186, *P* < 0.05 and β = 77.990, *P* < 0.01, respectively, Supplementary Table 12).

A trend was also observed for increased FCN2 with high CSF Aβ_42_ in the MCI group, by both logistic and linear regression (β = 0.315, *P* = 0.058 and β = -42.573, *P* = 0.051, respectively, Supplementary Table 11), replicating the association of FCN2 with CSF Tau/Aβ_42_ in the Amsterdam Dementia Cohort and with PET amyloid in MCI in the GE067005 study.

#### Univariate Statistical Analysis of Plasma Proteins in Relation to CSF tTau and pTau

We next assessed the relationship of the plasma proteins with CSF tTau and pTau in all AD, MCI and CTL subjects combined (*n* = 494). In the high CSF tTau pathology group, clusterin and complement C4B were nominally significantly increased (β = 0.379, *P* < 0.01 and β = 0.284, *P* < 0.05, respectively, Supplementary Table 13) and Complement C4 nominally significantly decreased (β = -0.269, *P* < 0.05, Supplementary Table 13) by logistic regression. Associations with CSF tTau by linear regression were also observed for clusterin (β = 42.089, *P* < 0.05, Supplementary Table 13), complement C4 (β = -56.915, *P* < 0.01, Supplementary Table 13) and Ig kappa chain C region (β = -37.292, *P* < 0.05, Supplementary Table 13). Logistic regression showed a nominally significant increase in clusterin (β = 0.284, *P* < 0.05) and nominally significant reduction of C4 (β = -0.318, *P* < 0.01) in association in the high CSF pTau group (Supplementary Table 14). Associations of clusterin and C4 with CSF pTau were also observed by linear regression (β = 5.083, *P* < 0.05, and β = -5.267, *P* < 0.05, respectively, Supplementary Table 14). AGP was also nominally significantly associated with CSF pTau by linear regression (β = -4.971, *P* < 0.05, Supplementary Table 14).

Within the AD group no proteins were associated with CSF tTau (Supplementary Table 15), whilst FCN2 was nominally significantly reduced in the high CSF pTau pathology group by logistic regression (β = -0.678, *P* < 0.05, Supplementary Table 18). In the MCI group, clusterin was nominally significantly increased in the high CSF tTau group (β = 0.409, *P* < 0.05, Supplementary Table 16) and C4 was nominally significantly associated with CSF tTau by both logistic and linear regression (β = -0.441, *P* < 0.05 and β = -47.627, *P* < 0.05, respectively, Supplementary Table 16). C4 was also decreased in the high CSF pTau pathology group (β = -0.468, *P* < 0.05, Supplementary Table 19). In the control group C4B was nominally significantly increased in the high CSF tTau group (β = 1.771, *P* < 0.05, Supplementary Table 17), whilst there were no significant associations CSF pTau (Supplementary Table 20).

#### Classification Analysis for the Prediction of Amyloid Status in the EMIF500 Cohort (CSF Aβ_42_ Group)

After excluding subjects with missing data, the classification analysis was performed on 96 subjects from the EMIF 500 study. These subjects were roughly evenly split between high and low amyloid groups (low CSF Aβ_42,_ *n* = 42; high CSF Aβ_42,_ *n* = 54). The minimal protein set with optimal AUC characteristics for prediction of CSF Aβ_42_ amyloid was assessed by SVM combined with LASSO and performance was assessed using 100 repeats of 10-fold cross validation. Five proteins (A1AT HAGP, Ig Kappa Chain C region, PEDF, and RANTES) formed the minimal protein panel for classifying amyloid positivity and achieved modest accuracy (AUC = 0.67 (Figure 3), PPV = 0.47, NPV = 0.47, sensitivity = 0.55, and specificity = 0.41).

## Discussion

In this study, we describe the discovery, replication and validation of plasma protein biomarkers relating to AD pathology and progression using an amyloid and tau pathology endophenotype based design. The success of recent clinical trials of disease-modifying therapies targeting Aβ have been hampered by lack of brain amyloid pathology in clinically diagnosed AD participants (Salloway et al., 2014) and in future it is likely that many trials of potential disease modifying agents will utilize biomarkers such as CSF amyloid and tau and amyloid PET measures of pathology in participant selection. However, such markers are relatively invasive and demanding of resource and participant commitment. Identifying participants with pathology using such methods in the preclinical and prodromal phase of disease is difficult and results in high screen failure in clinical trials. The cost of such screen failure is high, often prohibitively so. Even a modest reduction in screen failure rates would represent a major advance, certainly reducing costs and potentially accelerating speed of recruitment to such trials of disease modifying agents. Blood based biomarkers that can detect individuals likely to harbor AD pathology may therefore provide a cost-effective aid in triaging potential trial participants for PET or CSF based tests, helping to reduce screen failure, patient burden and costs. Moreover, a minimally invasive and cost-effective biomarker of AD pathology may help facilitate trials where repeated testing and monitoring of pathology is required.

We have first used LC-MS/MS to identify twenty-five plasma protein biomarkers of CSF Tau/Aβ_42_, and then replicated by ELISA the nominal association with CSF Tau/Aβ_42_ of three of the eight proteins subjected to further analysis; FCN2, ApoC-IV and FGB chain. Lack of ELISA-based replication of the remaining five proteins may be in part due to key platform differences. Mass spectrometry involves the analysis of peptides resulting from denatured protein, whilst ELISA measures intact protein, or more precisely the region of the intact protein where the epitope recognized by the antibody resides. It is possible therefore that protein region differences in turnover and abundance and other post-translational modifications including phosphorylation, glycosylation, and other changes may impact these results. Further replication studies examining protein region-specific abundance would therefore be needed to confirm the association of the proteins with CSF Tau/Aβ_42_.

Pathway analysis of the LC-MS/MS proteomic data revealed the significant differential regulation of a number of lipid-related pathways in association with CSF Tau/Aβ_42_ pathology. This is in line with the high representation of apolipoproteins we observed significantly associated with CSF measures of pathology. Furthermore, these findings are in agreement with the alteration to brain lipid metabolism observed in AD (Bales, 2010) and with pathway analysis of genomic association data (Jones et al., 2010). The proteins significantly associated with CSF measures of AD pathology were also shown to be associated with three disease groups using informatics approaches; familial hypercholesterolemia, brain diseases and metabolic bone disorder. These findings support the previously documented association of cholesterol and hypercholesterolemia with AD pathology (Refolo et al., 2000; Gibson Wood et al., 2003) and the association of osteoporosis with risk of developing AD (Zhou et al., 2014).

The most striking finding from all three phases of the current study – discovery, replication and validation – is the nominal association of FCN2 with AD pathology measures. This finding was consistent across measures used to assess pathology (CSF and PET) and using independent or orthogonal protein assay technologies (mass spectrometry and ELISA). Moreover, the association of FCN2 with CSF Aβ_42_ but not CSF Tau or pTau in the Amsterdam Dementia Cohort, suggests that this is driven by an association with amyloid pathology, in line with the replication results we report here from the GE067-005 PET amyloid study. However, in the EMIF500 cohort we do see a significant association of FCN2 with pTau. However, this association is only found in AD subjects, whereas earlier in the disease course we see a trend toward a significant relationship between FCN2 and CSF Aβ_42_ in the MCI subjects from this cohort. Given the studies that suggest CSF biomarkers are more sensitive to early change than PET biomarkers (Toledo et al., 2015) it might be that change in FCN2 measures could follow change in amyloid pathology particularly in the preclinical and prodromal stages of the disease. Such a hypothesis emphasizes the need for longitudinal studies of biomarkers in AD.

To our knowledge this is the first study to identify and validate FCN2 as a biomarker of AD pathology. Ficolins and mannose-binding lectins (MBL) are both activators of the lectin complement pathway (Fujita et al., 2004) and CSF MBL levels have been shown to be reduced in AD (Lanzrein et al., 1998). Another member of the ficolin family; ficolin-3 (FCN3), which shares approximately 50% amino acid sequence homology with FCN2 (Kilpatrick and Chalmers, 2012), is also associated with insulin resistance and diabetes (Li et al., 2008; Chen et al., 2012; Zhang et al., 2016). The association of the ficolin family with diabetes is interesting given that the relationship between diabetes and AD is well documented (Janson et al., 2004; Talbot et al., 2012).

Whilst Aβ40 and AopC4 were included in minimal protein panel with optimal accuracy for classifying high [^18^F]-flutemetamol PET amyloid from low [^18^F]-flutemetamol PET amyloid subjects, the accuracy of the 6-protein classifier was only modest (AUC = 0.69). Given that the proteins measured in this study included those that were previously identified as markers of other AD related measures [including cognitive decline, CSF Tau/Aβ and brain atrophy (Hye et al., 2014; Sattlecker et al., 2014)] they may not necessarily be specific to the load of fibrillised amyloid deposits in brain. Moreover, changes in CSF Aβ and tau, PET amyloid, MRI measures of brain atrophy and clinical measures of decline are all detectable at different stages of disease (Jack et al., 2013). We would therefore not necessarily expect all of these proteins to be related to amyloid at the MCI stage. In order to evaluate the biomarker utility of these proteins further, testing in larger independent cohorts with measures relating to various aspects of disease pathology and stage would be useful.

In the GE067-005 study cohort associations of Apo(a), ApoA-I, Ceruloplasmin and PPY with MCI conversion to AD were observed, and increased levels of ApoA-I were also tending toward an association with high [^18^F]-flutemetamol PET. All four proteins have previously been suggested as putative blood markers related to AD. For example, Apo(a) has previously been shown to be increased with high PiB PET amyloid (Ashton et al., 2015). Whilst increased plasma ApoA-1 in association with cognitive decline (Thambisetty et al., 2010; Song et al., 2012) and brain atrophy (Hye et al., 2014) have been observed. Decreased ApoA-1 levels in AD versus controls (Liu et al., 2006; Shih et al., 2014) and in association with increased risk of clinical progression to MCI and AD (Slot et al., 2017) and PiB PET amyloid (Ashton et al., 2015; Westwood et al., 2016) have also been shown. Moreover, ApoA-1 has been implicated in amyloid pathology, binding Aβ and protecting hippocampal neuronal cultures from Aβ-induced neurodegeneration (Paula-Lima et al., 2009).

In this study we use a range of proteomics approaches, building on previous studies from our group and others that have indicated a protein signature in blood that differentiates disease from non-disease and measures correlates with ‘endophenotypes’ of that disease state, as previously reviewed (Thambisetty and Lovestone, 2010; Baird et al., 2015; Shi et al., 2017). Others have taken a more direct route to blood biomarkers of AD, seeking to measure amyloid directly. Early studies using immunocapture were largely unsuccessful in identifying a marker methodology that predicted brain amyloid and was stable across studies and disease phases. More recently, using mass spectrometry and immunocapture with novel antibodies studies have reported excellent power in predicting brain amyloid load (Pesini et al., 2012; Ovod et al., 2017; Nakamura et al., 2018). However, whilst these studies show enormous potential, in some cases the methods are not yet suitable for application at scale, in large multi-site studies, require bespoke sample collection protocols and are likely to be resource intensive. These studies have however, clearly confirmed the findings of early biomarker studies that there is a signature in blood that reflects disease pathology. The use of multiplexed immunocapture as we describe here is a low-cost technology, readily applicable in the context of very large multi-center trials and therefore may have real world utility alongside any other approach to blood based biomarkers being developed.

In conclusion, in this study we have identified a number of proteins that are associated with CSF Aβ_42_/tau pathology. We identified and replicated FCN2 as a novel biomarker of both CSF and PET measures of AD pathology in an independent cohort and by independent proteomic platforms. Furthermore, we find an association of C4 with [^18^F]-flutemetamol PET amyloid and four proteins; Apo(a), ApoA-I, Ceruloplasmin and PPY with MCI conversion to AD, building upon previous findings of their relationship with AD and amyloid pathology. These results would suggest a biologically relevant role for these proteins in AD. Further analysis of the potential of these proteins as a biomarker of AD pathology and progression, in combination with other proteins or multimodal measures, and in larger independent cohorts will be essential. Such a blood-based biomarker could be of value as a triaging tool for PET and CSF based tests and hence aid in recruitment to clinical trials of disease modifying treatments.

## Data Availability Statement

The datasets for this manuscript will be made available by the authors to qualified researchers upon reasonable request. Requests to access the datasets should be directed to the corresponding author.

## Ethics Statement

This study was carried out in accordance with the recommendations of METC of VU University Medical Center for the Amsterdam Dementia Cohort, and the medical ethics committee at each site for the GE067-005 and EMIF 500 cohorts (Supplementary Tables 21 and 22, respectively) with written informed consent from all subjects. All subjects gave written informed consent in accordance with the Declaration of Helsinki. The protocol was approved by the METC of VU University Medical Center for the Amsterdam Dementia Cohort and the medical ethics medical ethics committee at each site for the GE067-005 and EMIF 500 cohorts (Supplementary Table 21 and 22, respectively).

## Author Contributions

AB, SL, AH, CB, MW, CTH, SB, VN, PS, and CT contributed to study design. BN, MZ, KD, SB, VN, WvdF, DG, LP, AL, PV, PS, and CT contributed to sample selection and provision. AB, SW, AH, SA, and NA were responsible for data acquisition. AB, SW, AH, SK, AN-H, BL, and DN carried out data analysis and interpretation. SW and AB drafted the manuscript. All authors revised the manuscript.

## Conflict of Interest Statement

SL is named as an inventor on biomarker intellectual property patent protected by Proteome Sciences and King’s College London. PS has received grant funding from GE Healthcare (paid to the institution). The remaining authors declare that the research was conducted in the absence of any commercial or financial relationships that could be construed as a potential conflict of interest.

## Footnotes

1 www.emif.eu

2 https://clinicaltrials.gov/ct2/show/NCT01028053

3 http://www.matrixscience.com
