# Supplementary material for: Plasma Protein Biomarkers for the Prediction of CSF Amyloid and Tau and [18F]-Flutemetamol PET Scan Result
Source: Front Aging Neurosci. 2018 Dec 11;10:409. doi: 10.3389/fnagi.2018.00409 (PMC6297196; doi:10.3389/fnagi.2018.00409)
Supplement: Supplementary file 2 [file Data_Sheet_2.docx]

**Supplementary Table 7. Associations of proteins with [18F] Flutemetamol PET amyloid.** Abbreviations: Uniprot, universal protein resource. *Nominally significant *P* < 0.05.

| **UniProt ID** | **Protein Name** | **Logistic Regression** | | | **Linear Regression** | | |
| --- | --- | --- | --- | --- | --- | --- | --- |
|  |  | **β** | ***P* value** | ***q* value** | **β** | ***P* value** | ***q* value** |
| P02763 | Alpha-1-acid glycoprotein 1 | 0.076 | 0.766 | 0.913 | -0.036 | 0.419 | 0.732 |
| P01009 | Alpha-1-antitrypsin | -0.036 | 0.872 | 0.927 | 0.021 | 0.560 | 0.736 |
| P01023 | Alpha-2-macroglobulin (ELISA) | -0.050 | 0.832 | 0.913 | -0.003 | 0.940 | 0.996 |
| P01023 | Alpha-2-macroglobulin (xMAP) | 0.141 | 0.493 | 0.882 | 0.024 | 0.478 | 0.735 |
| / | Amyloid-beta 40 | 0.368 | 0.092 | 0.697 | 0.065 | 0.079 | 0.603 |
| P02647 | Apolipoprotein A-I | 0.498 | 0.054 | 0.610 | 0.074 | 0.052 | 0.603 |
| P02656 | Apolipoprotein C-III | 0.247 | 0.256 | 0.724 | 0.057 | 0.099 | 0.603 |
| P55056 | Apolipoprotein C-IV | 0.618 | 0.116 | 0.697 | 0.109 | 0.072 | 0.603 |
| P02649 | Apolipoprotein E | -0.063 | 0.763 | 0.913 | 0.000 | 0.996 | 0.996 |
| P08519 | Apolipoprotein(a) | 0.101 | 0.677 | 0.913 | 0.010 | 0.789 | 0.902 |
| P61769 | Beta-2-microglobulin | 0.324 | 0.162 | 0.724 | 0.032 | 0.399 | 0.732 |
| P23560 | Brain-derived neurotrophic factor | 0.053 | 0.821 | 0.913 | 0.000 | 0.992 | 0.996 |
| P07339 | Cathepsin D | -0.295 | 0.185 | 0.724 | -0.045 | 0.199 | 0.603 |
| P00450 | Ceruloplasmin | -0.064 | 0.766 | 0.913 | -0.043 | 0.248 | 0.603 |
| P10909 | Clusterin | 0.295 | 0.244 | 0.724 | 0.028 | 0.497 | 0.735 |
| / | Complement component 4 | 0.750 | 0.012* | 0.268 | 0.079 | 0.046* | 0.603 |
| P08603 | Complement factor H | 0.026 | 0.902 | 0.929 | 0.048 | 0.170 | 0.603 |
| Q03591 | Complement factor H-related protein 1 | -0.376 | 0.428 | 0.882 | -0.099 | 0.182 | 0.603 |
| P02741 | C-reactive protein | -0.079 | 0.716 | 0.913 | -0.007 | 0.855 | 0.938 |
| P01034 | Cystatin C | 0.322 | 0.123 | 0.697 | 0.029 | 0.410 | 0.732 |
| P02679 | Fibrinogen gamma chain | -0.014 | 0.956 | 0.956 | -0.020 | 0.644 | 0.812 |
| Q15485 | Ficolin-2 | 0.580 | 0.016* | 0.268 | 0.049 | 0.195 | 0.603 |
| P01834 | Ig kappa chain C region | -0.108 | 0.616 | 0.913 | -0.046 | 0.232 | 0.603 |
| P05362 | Intercellular adhesion molecule 1 | -0.140 | 0.489 | 0.882 | -0.013 | 0.704 | 0.855 |
| P13591 | Neural cell adhesion molecule 1 | 0.196 | 0.369 | 0.882 | 0.036 | 0.316 | 0.717 |
| P09104 | Neuron-specific enolase | -0.083 | 0.739 | 0.913 | -0.023 | 0.563 | 0.736 |
| P01298 | Pancreatic prohormone | -0.091 | 0.690 | 0.913 | 0.025 | 0.536 | 0.736 |
| P36955 | Pigment epithelium-derived factor | 0.520 | 0.246 | 0.724 | 0.100 | 0.193 | 0.603 |
| P05121 | Plasminogen activator inhibitor 1 | -0.277 | 0.305 | 0.799 | -0.051 | 0.248 | 0.603 |
| P13501 | RANTES | -0.165 | 0.461 | 0.882 | -0.027 | 0.452 | 0.732 |
| P02787 | Serotransferrin | 0.100 | 0.689 | 0.913 | -0.034 | 0.439 | 0.732 |
| P02743 | Serum amyloid P-component | 0.337 | 0.255 | 0.724 | 0.068 | 0.174 | 0.603 |
| P02766 | Transthyretin | 0.148 | 0.490 | 0.882 | 0.030 | 0.406 | 0.732 |
| P19320 | Vascular cell adhesion protein 1 | 0.070 | 0.783 | 0.913 | 0.011 | 0.796 | 0.902 |

**Supplementary Table 8. Associations of proteins with conversion from MCI to AD.** Abbreviations: Uniprot, universal protein resource; MCI, mild cognitive impairment; AD, Alzheimer's disease. *Nominally significant *P* < 0.05.

| **UniProt ID** | **Protein Name** | **Logistic Regression** | | |
| --- | --- | --- | --- | --- |
|  |  | **β** | ***P* value** | ***q* value** |
| P02763 | Alpha-1-acid glycoprotein 1 | -0.343 | 0.177 | 0.644 |
| P01009 | Alpha-1-antitrypsin | 0.302 | 0.123 | 0.524 |
| P01023 | Alpha-2-macroglobulin (ELISA) | 0.241 | 0.305 | 0.647 |
| P01023 | Alpha-2-macroglobulin (xMAP) | 0.360 | 0.077 | 0.436 |
| / | Amyloid-beta 40 | -0.336 | 0.101 | 0.489 |
| P02647 | Apolipoprotein A-I | 0.631 | 0.014* | 0.361 |
| P02656 | Apolipoprotein C-III | 0.138 | 0.497 | 0.826 |
| P55056 | Apolipoprotein C-IV | -0.048 | 0.890 | 0.946 |
| P02649 | Apolipoprotein E | 0.097 | 0.624 | 0.826 |
| P08519 | Apolipoprotein(a) | -0.476 | 0.049* | 0.419 |
| P61769 | Beta-2-microglobulin | -0.042 | 0.839 | 0.946 |
| P23560 | Brain-derived neurotrophic factor | 0.123 | 0.591 | 0.826 |
| P07339 | Cathepsin D | 0.229 | 0.255 | 0.644 |
| P00450 | Ceruloplasmin | -0.526 | 0.021* | 0.361 |
| P10909 | Clusterin | -0.033 | 0.888 | 0.946 |
| / | Complement component 4 | 0.012 | 0.960 | 0.988 |
| P08603 | Complement factor H | -0.003 | 0.988 | 0.988 |
| Q03591 | Complement factor H-related protein 1 | 0.321 | 0.438 | 0.784 |
| P02741 | C-reactive protein | -0.232 | 0.284 | 0.644 |
| P01034 | Cystatin C | 0.156 | 0.426 | 0.784 |
| P02679 | Fibrinogen gamma chain | 0.271 | 0.278 | 0.644 |
| Q15485 | Ficolin-2 | 0.262 | 0.228 | 0.644 |
| P01834 | Ig kappa chain C region | -0.099 | 0.632 | 0.826 |
| P05362 | Intercellular adhesion molecule 1 | 0.210 | 0.279 | 0.644 |
| P13591 | Neural cell adhesion molecule 1 | 0.249 | 0.213 | 0.644 |
| P09104 | Neuron-specific enolase | 0.099 | 0.676 | 0.832 |
| P01298 | Pancreatic prohormone | -0.456 | 0.048* | 0.419 |
| P36955 | Pigment epithelium-derived factor | 0.391 | 0.371 | 0.741 |
| P05121 | Plasminogen activator inhibitor 1 | 0.521 | 0.066 | 0.436 |
| P13501 | RANTES | 0.086 | 0.685 | 0.832 |
| P02787 | Serotransferrin | -0.165 | 0.530 | 0.826 |
| P02743 | Serum amyloid P-component | -0.136 | 0.632 | 0.826 |
| P02766 | Transthyretin | -0.069 | 0.742 | 0.870 |
| P19320 | Vascular cell adhesion protein 1 | -0.133 | 0.594 | 0.826 |

**Supplementary Table 9. Associations of proteins with CSF Aβ_42_ in the EMIF study.** Abbreviations: Uniprot, universal protein resource. *Nominally significant *P* < 0.05.

| **UniProt ID** | **Protein Name** | **Logistic Regression** | | | **Linear Regression** | | |
| --- | --- | --- | --- | --- | --- | --- | --- |
|  |  | **β** | ***P* value** | ***q* value** | **β** | ***P* value** | ***q* value** |
| P02763 | Alpha-1-acid glycoprotein 1 | -0.121 | 0.369 | 0.685 | 19.104 | 0.300 | 0.966 |
| P01009 | Alpha-1-antitrypsin | -0.248 | 0.024* | 0.309 | 31.690 | 0.034* | 0.716 |
| P01023 | Alpha-2-macroglobulin | 0.031 | 0.836 | 0.978 | 0.099 | 0.996 | 0.996 |
| P02647 | Apolipoprotein A-I | 0.162 | 0.219 | 0.575 | -4.433 | 0.790 | 0.976 |
| P02656 | Apolipoprotein C-III | -0.042 | 0.854 | 0.978 | 17.070 | 0.564 | 0.966 |
| P55056 | Apolipoprotein C-IV | 0.021 | 0.875 | 0.978 | -6.700 | 0.706 | 0.966 |
| P08519 | Apolipoprotein(a) | -0.097 | 0.401 | 0.685 | 5.831 | 0.709 | 0.966 |
| P10909 | Clusterin | 0.278 | 0.033* | 0.309 | -14.413 | 0.402 | 0.966 |
| P0C0L5 | Complement C4-B | 0.036 | 0.784 | 0.978 | 0.493 | 0.976 | 0.996 |
| / | Complement component 4 | -0.151 | 0.215 | 0.575 | 9.173 | 0.573 | 0.966 |
| Q03591 | Complement factor H-related protein 1 | -0.184 | 0.313 | 0.657 | 12.454 | 0.604 | 0.966 |
| P01034 | Cystatin C | 0.011 | 0.932 | 0.978 | 8.818 | 0.607 | 0.966 |
| P02679 | Fibrinogen gamma chain | -0.213 | 0.074 | 0.309 | 16.612 | 0.294 | 0.966 |
| Q15485 | Ficolin-2 | 0.216 | 0.055 | 0.309 | -22.985 | 0.121 | 0.845 |
| P01860 | Ig gamma-3 chain C region | -0.088 | 0.424 | 0.685 | 16.889 | 0.251 | 0.966 |
| P01834 | Ig kappa chain C region | -0.152 | 0.169 | 0.575 | 24.957 | 0.101 | 0.845 |
| P05362 | Intercellular adhesion molecule 1 | 0.010 | 0.930 | 0.978 | 1.308 | 0.933 | 0.996 |
| P36955 | Pigment epithelium-derived factor | -0.271 | 0.272 | 0.634 | 23.027 | 0.476 | 0.966 |
| P13501 | RANTES | -0.222 | 0.066 | 0.309 | 5.647 | 0.736 | 0.966 |
| P02787 | Serotransferrin | 0.078 | 0.558 | 0.838 | 6.304 | 0.719 | 0.966 |
| P02766 | Transthyretin | -0.003 | 0.983 | 0.983 | 2.787 | 0.875 | 0.996 |

**Supplementary Table 10. Associations of proteins with CSF Aβ_42_ in AD in the EMIF study.** Abbreviations: Uniprot, universal protein resource. Nominally significant *P* < 0.05* *P* < 0.01**.

| **UniProt ID** | **Protein Name** | **Logistic Regression** | | | **Linear Regression** | | |
| --- | --- | --- | --- | --- | --- | --- | --- |
|  |  | **β** | ***P* value** | ***q* value** | **β** | ***P* value** | ***q* value** |
| P02763 | Alpha-1-acid glycoprotein 1 | -0.414 | 0.490 | 0.791 | 24.050 | 0.172 | 0.904 |
| P01009 | Alpha-1-antitrypsin | 0.170 | 0.793 | 0.876 | 7.284 | 0.599 | 0.982 |
| P01023 | Alpha-2-macroglobulin | -1.858 | 0.057 | 0.596 | 6.386 | 0.750 | 0.982 |
| P02647 | Apolipoprotein A-I | -0.117 | 0.882 | 0.882 | 14.412 | 0.330 | 0.977 |
| P02656 | Apolipoprotein C-III | -0.411 | 0.591 | 0.816 | 20.313 | 0.419 | 0.977 |
| P55056 | Apolipoprotein C-IV | -0.568 | 0.306 | 0.649 | 0.673 | 0.968 | 0.982 |
| P08519 | Apolipoprotein(a) | 1.220 | 0.204 | 0.649 | -3.571 | 0.807 | 0.982 |
| P10909 | Clusterin | 0.748 | 0.276 | 0.649 | -3.087 | 0.860 | 0.982 |
| P0C0L5 | Complement C4-B | 0.953 | 0.307 | 0.649 | -25.368 | 0.108 | 0.897 |
| / | Complement component 4 | 0.090 | 0.863 | 0.882 | 8.807 | 0.547 | 0.982 |
| Q03591 | Complement factor H-related protein 1 | 0.966 | 0.203 | 0.649 | -27.189 | 0.219 | 0.921 |
| P01034 | Cystatin C | 0.358 | 0.555 | 0.816 | -11.829 | 0.494 | 0.982 |
| P02679 | Fibrinogen gamma chain | -0.122 | 0.727 | 0.848 | -2.384 | 0.866 | 0.982 |
| Q15485 | Ficolin-2 | 0.587 | 0.275 | 0.649 | 0.930 | 0.945 | 0.982 |
| P01860 | Ig gamma-3 chain C region | 0.219 | 0.622 | 0.816 | 1.231 | 0.926 | 0.982 |
| P01834 | Ig kappa chain C region | -0.157 | 0.711 | 0.848 | 5.331 | 0.713 | 0.982 |
| P05362 | Intercellular adhesion molecule 1 | -0.379 | 0.318 | 0.649 | -12.470 | 0.388 | 0.977 |
| P36955 | Pigment epithelium-derived factor | 1.076 | 0.371 | 0.649 | 0.616 | 0.982 | 0.982 |
| P13501 | RANTES | -1.192 | 0.009** | 0.181 | 35.759 | 0.015* | 0.312 |
| P02787 | Serotransferrin | -0.982 | 0.106 | 0.649 | 24.622 | 0.128 | 0.897 |
| P02766 | Transthyretin | -0.457 | 0.347 | 0.649 | 15.218 | 0.334 | 0.977 |

**Supplementary Table 11. Associations of proteins with CSF Aβ_42_ in MCI in the EMIF study.** Abbreviations: Uniprot, universal protein resource. **Nominally significant *P* < 0.01.

| **UniProt ID** | **Protein Name** | **Logistic Regression** | | | **Linear Regression** | | |
| --- | --- | --- | --- | --- | --- | --- | --- |
|  |  | **β** | ***P* value** | ***q* value** | **β** | ***P* value** | ***q* value** |
| P02763 | Alpha-1-acid glycoprotein 1 | -0.315 | 0.108 | 0.564 | 37.791 | 0.154 | 0.851 |
| P01009 | Alpha-1-antitrypsin | -0.202 | 0.225 | 0.724 | 1.731 | 0.937 | 0.988 |
| P01023 | Alpha-2-macroglobulin | 0.140 | 0.501 | 0.813 | -10.667 | 0.721 | 0.988 |
| P02647 | Apolipoprotein A-I | 0.137 | 0.453 | 0.813 | -8.386 | 0.732 | 0.988 |
| P02656 | Apolipoprotein C-III | 0.009 | 0.981 | 0.981 | -2.768 | 0.951 | 0.988 |
| P55056 | Apolipoprotein C-IV | 0.066 | 0.731 | 0.813 | -0.417 | 0.988 | 0.988 |
| P08519 | Apolipoprotein(a) | -0.075 | 0.650 | 0.813 | -20.092 | 0.371 | 0.988 |
| P10909 | Clusterin | 0.062 | 0.727 | 0.813 | 3.781 | 0.876 | 0.988 |
| P0C0L5 | Complement C4-B | 0.112 | 0.566 | 0.813 | 4.176 | 0.868 | 0.988 |
| / | Complement component 4 | -0.104 | 0.574 | 0.813 | -16.052 | 0.524 | 0.988 |
| Q03591 | Complement factor H-related protein 1 | -0.092 | 0.735 | 0.813 | 3.524 | 0.922 | 0.988 |
| P01034 | Cystatin C | -0.143 | 0.414 | 0.813 | 26.657 | 0.266 | 0.931 |
| P02679 | Fibrinogen gamma chain | -0.097 | 0.583 | 0.813 | 4.752 | 0.843 | 0.988 |
| Q15485 | Ficolin-2 | 0.315 | 0.058 | 0.564 | -42.573 | 0.051 | 0.531 |
| P01860 | Ig gamma-3 chain C region | 0.035 | 0.831 | 0.872 | 12.213 | 0.574 | 0.988 |
| P01834 | Ig kappa chain C region | -0.185 | 0.241 | 0.724 | 25.016 | 0.251 | 0.931 |
| P05362 | Intercellular adhesion molecule 1 | -0.277 | 0.098 | 0.564 | 66.327 | 0.003** | 0.064 |
| P36955 | Pigment epithelium-derived factor | -0.329 | 0.392 | 0.813 | -5.074 | 0.923 | 0.988 |
| P13501 | RANTES | -0.264 | 0.144 | 0.604 | 6.622 | 0.785 | 0.988 |
| P02787 | Serotransferrin | 0.209 | 0.303 | 0.796 | -16.264 | 0.520 | 0.988 |
| P02766 | Transthyretin | 0.377 | 0.070 | 0.564 | -37.590 | 0.162 | 0.851 |

**Supplementary Table 12. Associations of proteins with CSF Aβ_42_ in CTL in the EMIF study.** Abbreviations: Uniprot, universal protein resource. Nominally significant *P* < 0.05*, *P* < 0.01**.

| **UniProt ID** | **Protein Name** | **Logistic Regression** | | | **Linear Regression** | | |
| --- | --- | --- | --- | --- | --- | --- | --- |
|  |  | **β** | ***P* value** | ***q* value** | **β** | ***P* value** | ***q* value** |
| P02763 | Alpha-1-acid glycoprotein 1 | 0.377 | 0.390 | 0.745 | -21.468 | 0.566 | 0.901 |
| P01009 | Alpha-1-antitrypsin | -1.186 | 0.038* | 0.264 | 77.990 | 0.008** | 0.163 |
| P01023 | Alpha-2-macroglobulin | 0.806 | 0.128 | 0.540 | -12.800 | 0.781 | 0.901 |
| P02647 | Apolipoprotein A-I | 0.227 | 0.546 | 0.807 | 8.983 | 0.796 | 0.901 |
| P02656 | Apolipoprotein C-III | -0.686 | 0.315 | 0.708 | 69.018 | 0.285 | 0.835 |
| P55056 | Apolipoprotein C-IV | 0.070 | 0.847 | 0.875 | -1.265 | 0.971 | 0.971 |
| P08519 | Apolipoprotein(a) | -0.152 | 0.677 | 0.807 | 43.113 | 0.144 | 0.780 |
| P10909 | Clusterin | 0.174 | 0.693 | 0.807 | 36.050 | 0.318 | 0.835 |
| P0C0L5 | Complement C4-B | -0.057 | 0.875 | 0.875 | -14.612 | 0.690 | 0.901 |
| / | Complement component 4 | 0.132 | 0.721 | 0.807 | -39.935 | 0.186 | 0.780 |
| Q03591 | Complement factor H-related protein 1 | -1.435 | 0.028* | 0.264 | 82.901 | 0.082 | 0.780 |
| P01034 | Cystatin C | -0.218 | 0.564 | 0.807 | 23.949 | 0.499 | 0.901 |
| P02679 | Fibrinogen gamma chain | -1.003 | 0.024* | 0.264 | 45.054 | 0.177 | 0.780 |
| Q15485 | Ficolin-2 | -0.435 | 0.220 | 0.661 | 34.477 | 0.248 | 0.835 |
| P01860 | Ig gamma-3 chain C region | 0.143 | 0.691 | 0.807 | -1.848 | 0.956 | 0.971 |
| P01834 | Ig kappa chain C region | 0.178 | 0.569 | 0.807 | 17.375 | 0.581 | 0.901 |
| P05362 | Intercellular adhesion molecule 1 | 0.342 | 0.337 | 0.708 | -9.660 | 0.752 | 0.901 |
| P36955 | Pigment epithelium-derived factor | -1.339 | 0.216 | 0.661 | 57.752 | 0.367 | 0.856 |
| P13501 | RANTES | -0.499 | 0.275 | 0.708 | -17.754 | 0.601 | 0.901 |
| P02787 | Serotransferrin | 0.878 | 0.059 | 0.308 | -8.645 | 0.815 | 0.901 |
| P02766 | Transthyretin | -0.152 | 0.730 | 0.807 | 8.142 | 0.815 | 0.901 |

**Supplementary Table 13. Associations of proteins with CSF tTau in the EMIF study.** Abbreviations: Uniprot, universal protein resource. Nominally significant **P* < 0.05, ***P* < 0.01.

| **UniProt ID** | **Protein Name** | **Logistic Regression** | | | **Linear Regression** | | |
| --- | --- | --- | --- | --- | --- | --- | --- |
|  |  | **β** | ***P* value** | ***q* value** | **β** | ***P* value** | ***q* value** |
| P02763 | Alpha-1-acid glycoprotein 1 | -0.183 | 0.189 | 0.661 | -34.459 | 0.078 | 0.377 |
| P01009 | Alpha-1-antitrypsin | -0.156 | 0.169 | 0.661 | -6.016 | 0.713 | 0.861 |
| P01023 | Alpha-2-macroglobulin | -0.007 | 0.965 | 0.965 | -10.456 | 0.643 | 0.861 |
| P02647 | Apolipoprotein A-I | 0.125 | 0.323 | 0.853 | 12.201 | 0.474 | 0.829 |
| P02656 | Apolipoprotein C-III | 0.093 | 0.689 | 0.853 | -37.422 | 0.248 | 0.751 |
| P55056 | Apolipoprotein C-IV | 0.081 | 0.543 | 0.853 | 3.357 | 0.861 | 0.861 |
| P08519 | Apolipoprotein(a) | -0.091 | 0.443 | 0.853 | -4.658 | 0.777 | 0.861 |
| P10909 | Clusterin | 0.379 | 0.005** | 0.112 | 42.089 | 0.023* | 0.160 |
| P0C0L5 | Complement C4-B | 0.284 | 0.035* | 0.243 | 14.206 | 0.460 | 0.829 |
| / | Complement component 4 | -0.269 | 0.032* | 0.243 | -56.915 | 0.001** | 0.025 |
| Q03591 | Complement factor H-related protein 1 | -0.092 | 0.612 | 0.853 | 4.599 | 0.858 | 0.861 |
| P01034 | Cystatin C | 0.039 | 0.761 | 0.853 | -16.948 | 0.359 | 0.829 |
| P02679 | Fibrinogen gamma chain | -0.076 | 0.509 | 0.853 | -4.335 | 0.799 | 0.861 |
| Q15485 | Ficolin-2 | 0.046 | 0.671 | 0.853 | 13.605 | 0.392 | 0.829 |
| P01860 | Ig gamma-3 chain C region | -0.066 | 0.560 | 0.853 | -27.566 | 0.090 | 0.377 |
| P01834 | Ig kappa chain C region | -0.035 | 0.752 | 0.853 | -37.292 | 0.022* | 0.160 |
| P05362 | Intercellular adhesion molecule 1 | 0.155 | 0.179 | 0.661 | 8.012 | 0.635 | 0.861 |
| P36955 | Pigment epithelium-derived factor | -0.064 | 0.792 | 0.853 | -40.380 | 0.250 | 0.751 |
| P13501 | RANTES | -0.029 | 0.812 | 0.853 | 8.856 | 0.622 | 0.861 |
| P02787 | Serotransferrin | 0.109 | 0.404 | 0.853 | 3.441 | 0.853 | 0.861 |
| P02766 | Transthyretin | -0.078 | 0.552 | 0.853 | -15.447 | 0.420 | 0.829 |

**Supplementary Table 14. Associations of proteins with CSF pTau in the EMIF study.** Abbreviations: Uniprot, universal protein resource. Nominally significant **P* < 0.05, ***P* < 0.01.

| **UniProt ID** | **Protein Name** | **Logistic Regression** | | | **Linear Regression** | | |
| --- | --- | --- | --- | --- | --- | --- | --- |
|  |  | **β** | ***P* value** | ***q* value** | **β** | ***P* value** | ***q* value** |
| P02763 | Alpha-1-acid glycoprotein 1 | -0.197 | 0.138 | 0.485 | -4.971 | 0.027* | 0.187 |
| P01009 | Alpha-1-antitrypsin | -0.054 | 0.614 | 0.982 | 0.206 | 0.915 | 0.969 |
| P01023 | Alpha-2-macroglobulin | 0.001 | 0.992 | 0.998 | -0.880 | 0.747 | 0.969 |
| P02647 | Apolipoprotein A-I | 0.077 | 0.521 | 0.982 | -0.010 | 0.996 | 0.996 |
| P02656 | Apolipoprotein C-III | -0.126 | 0.567 | 0.982 | -4.746 | 0.211 | 0.672 |
| P55056 | Apolipoprotein C-IV | 0.012 | 0.924 | 0.998 | -0.462 | 0.836 | 0.969 |
| P08519 | Apolipoprotein(a) | -0.016 | 0.885 | 0.998 | 1.485 | 0.459 | 0.742 |
| P10909 | Clusterin | 0.284 | 0.028* | 0.299 | 5.083 | 0.018* | 0.187 |
| P0C0L5 | Complement C4-B | 0.039 | 0.752 | 0.987 | 2.188 | 0.299 | 0.672 |
| / | Complement component 4 | -0.318 | 0.009** | 0.180 | -5.267 | 0.011* | 0.187 |
| Q03591 | Complement factor H-related protein 1 | -0.229 | 0.188 | 0.564 | -1.059 | 0.726 | 0.969 |
| P01034 | Cystatin C | -0.116 | 0.358 | 0.939 | -2.050 | 0.343 | 0.672 |
| P02679 | Fibrinogen gamma chain | -0.167 | 0.139 | 0.485 | -1.733 | 0.381 | 0.672 |
| Q15485 | Ficolin-2 | -0.043 | 0.684 | 0.982 | 1.575 | 0.384 | 0.672 |
| P01860 | Ig gamma-3 chain C region | -0.011 | 0.918 | 0.998 | -2.135 | 0.261 | 0.672 |
| P01834 | Ig kappa chain C region | -0.053 | 0.622 | 0.982 | -2.980 | 0.122 | 0.641 |
| P05362 | Intercellular adhesion molecule 1 | 0.211 | 0.063 | 0.441 | 2.228 | 0.258 | 0.672 |
| P36955 | Pigment epithelium-derived factor | -0.088 | 0.702 | 0.982 | -0.400 | 0.923 | 0.969 |
| P13501 | RANTES | -0.195 | 0.111 | 0.485 | -0.423 | 0.841 | 0.969 |
| P02787 | Serotransferrin | 0.000 | 0.998 | 0.998 | -0.305 | 0.890 | 0.969 |
| P02766 | Transthyretin | -0.086 | 0.498 | 0.982 | -2.116 | 0.344 | 0.672 |

**Supplementary Table 15. Associations of proteins with CSF tTau in AD in the EMIF study.** Abbreviations: Uniprot, universal protein resource.

| **UniProt ID** | **Protein Name** | **Logistic Regression** | | | **Linear Regression** | | |
| --- | --- | --- | --- | --- | --- | --- | --- |
|  |  | **β** | ***P* value** | ***q* value** | **β** | ***P* value** | ***q* value** |
| P02763 | Alpha-1-acid glycoprotein 1 | -0.170 | 0.694 | 0.972 | -57.750 | 0.221 | 0.710 |
| P01009 | Alpha-1-antitrypsin | 0.407 | 0.385 | 0.856 | 37.029 | 0.304 | 0.710 |
| P01023 | Alpha-2-macroglobulin | 0.271 | 0.538 | 0.856 | -16.157 | 0.757 | 0.837 |
| P02647 | Apolipoprotein A-I | -0.076 | 0.829 | 0.977 | 8.330 | 0.807 | 0.847 |
| P02656 | Apolipoprotein C-III | -0.086 | 0.884 | 0.977 | -83.766 | 0.179 | 0.710 |
| P55056 | Apolipoprotein C-IV | 0.094 | 0.799 | 0.977 | -26.168 | 0.533 | 0.837 |
| P08519 | Apolipoprotein(a) | -0.223 | 0.525 | 0.856 | 13.083 | 0.705 | 0.837 |
| P10909 | Clusterin | 0.053 | 0.895 | 0.977 | -3.746 | 0.931 | 0.931 |
| P0C0L5 | Complement C4-B | 0.738 | 0.092 | 0.856 | 13.982 | 0.749 | 0.837 |
| / | Complement component 4 | 0.410 | 0.227 | 0.856 | -41.843 | 0.246 | 0.710 |
| Q03591 | Complement factor H-related protein 1 | 0.294 | 0.503 | 0.856 | 50.216 | 0.352 | 0.740 |
| P01034 | Cystatin C | -0.224 | 0.571 | 0.856 | -61.696 | 0.151 | 0.710 |
| P02679 | Fibrinogen gamma chain | 0.184 | 0.531 | 0.856 | 37.901 | 0.280 | 0.710 |
| Q15485 | Ficolin-2 | -0.467 | 0.132 | 0.856 | -19.803 | 0.558 | 0.837 |
| P01860 | Ig gamma-3 chain C region | -0.158 | 0.550 | 0.856 | -52.734 | 0.114 | 0.710 |
| P01834 | Ig kappa chain C region | 0.009 | 0.977 | 0.977 | -41.106 | 0.248 | 0.710 |
| P05362 | Intercellular adhesion molecule 1 | 0.016 | 0.958 | 0.977 | -22.569 | 0.532 | 0.837 |
| P36955 | Pigment epithelium-derived factor | 0.669 | 0.349 | 0.856 | -31.557 | 0.641 | 0.837 |
| P13501 | RANTES | -0.579 | 0.059 | 0.856 | -19.023 | 0.604 | 0.837 |
| P02787 | Serotransferrin | 0.298 | 0.492 | 0.856 | 33.236 | 0.412 | 0.786 |
| P02766 | Transthyretin | -0.214 | 0.559 | 0.856 | -46.082 | 0.241 | 0.710 |

**Supplementary Table 16. Associations of proteins with CSF tTau in MCI in the EMIF study.** Abbreviations: Uniprot, universal protein resource. Nominally significant **P* < 0.05.

| **UniProt ID** | **Protein Name** | **Logistic Regression** | | | **Linear Regression** | | |
| --- | --- | --- | --- | --- | --- | --- | --- |
|  |  | **β** | ***P* value** | ***q* value** | **β** | ***P* value** | ***q* value** |
| P02763 | Alpha-1-acid glycoprotein 1 | -0.265 | 0.182 | 0.689 | -27.492 | 0.241 | 0.557 |
| P01009 | Alpha-1-antitrypsin | -0.196 | 0.243 | 0.689 | -14.096 | 0.438 | 0.743 |
| P01023 | Alpha-2-macroglobulin | -0.054 | 0.792 | 0.846 | -5.019 | 0.839 | 0.881 |
| P02647 | Apolipoprotein A-I | 0.183 | 0.305 | 0.689 | 16.782 | 0.413 | 0.743 |
| P02656 | Apolipoprotein C-III | 0.404 | 0.270 | 0.689 | -18.376 | 0.634 | 0.881 |
| P55056 | Apolipoprotein C-IV | 0.278 | 0.151 | 0.689 | 25.305 | 0.265 | 0.557 |
| P08519 | Apolipoprotein(a) | -0.049 | 0.767 | 0.846 | -4.341 | 0.823 | 0.881 |
| P10909 | Clusterin | 0.409 | 0.033* | 0.350 | 38.573 | 0.059 | 0.557 |
| P0C0L5 | Complement C4-B | 0.239 | 0.219 | 0.689 | 25.287 | 0.259 | 0.557 |
| / | Complement component 4 | -0.441 | 0.022* | 0.350 | -47.627 | 0.023* | 0.477 |
| Q03591 | Complement factor H-related protein 1 | 0.064 | 0.806 | 0.846 | 7.626 | 0.800 | 0.881 |
| P01034 | Cystatin C | 0.141 | 0.420 | 0.735 | 2.824 | 0.889 | 0.889 |
| P02679 | Fibrinogen gamma chain | -0.102 | 0.553 | 0.774 | -28.633 | 0.153 | 0.557 |
| Q15485 | Ficolin-2 | 0.054 | 0.723 | 0.846 | 20.903 | 0.255 | 0.557 |
| P01860 | Ig gamma-3 chain C region | 0.153 | 0.341 | 0.689 | 13.961 | 0.460 | 0.743 |
| P01834 | Ig kappa chain C region | 0.138 | 0.361 | 0.689 | -23.011 | 0.212 | 0.557 |
| P05362 | Intercellular adhesion molecule 1 | 0.049 | 0.758 | 0.846 | -4.939 | 0.797 | 0.881 |
| P36955 | Pigment epithelium-derived factor | -0.217 | 0.550 | 0.774 | -25.940 | 0.548 | 0.822 |
| P13501 | RANTES | -0.024 | 0.890 | 0.890 | 26.113 | 0.196 | 0.557 |
| P02787 | Serotransferrin | 0.317 | 0.099 | 0.689 | 7.729 | 0.717 | 0.881 |
| P02766 | Transthyretin | 0.134 | 0.494 | 0.774 | 30.818 | 0.175 | 0.557 |

**Supplementary Table 17. Associations of proteins with CSF tTau in CTL in the EMIF study.** Abbreviations: Uniprot, universal protein resource. Nominally significant **P* < 0.05.

| **UniProt ID** | **Protein Name** | **Logistic Regression** | | | **Linear Regression** | | |
| --- | --- | --- | --- | --- | --- | --- | --- |
|  |  | **β** | ***P* value** | ***q* value** | **β** | ***P* value** | ***q* value** |
| P02763 | Alpha-1-acid glycoprotein 1 | 0.157 | 0.758 | 0.838 | -13.058 | 0.332 | 0.796 |
| P01009 | Alpha-1-antitrypsin | -0.019 | 0.962 | 0.962 | 8.588 | 0.422 | 0.796 |
| P01023 | Alpha-2-macroglobulin | -0.800 | 0.331 | 0.665 | -8.660 | 0.621 | 0.796 |
| P02647 | Apolipoprotein A-I | 0.448 | 0.443 | 0.665 | -11.836 | 0.362 | 0.796 |
| P02656 | Apolipoprotein C-III | -1.304 | 0.189 | 0.665 | 9.071 | 0.695 | 0.796 |
| P55056 | Apolipoprotein C-IV | -0.324 | 0.571 | 0.722 | 0.712 | 0.955 | 0.955 |
| P08519 | Apolipoprotein(a) | 0.238 | 0.584 | 0.722 | 15.894 | 0.144 | 0.796 |
| P10909 | Clusterin | 0.874 | 0.272 | 0.665 | 7.883 | 0.551 | 0.796 |
| P0C0L5 | Complement C4-B | 1.711 | 0.042* | 0.665 | 3.882 | 0.758 | 0.796 |
| / | Complement component 4 | 0.525 | 0.342 | 0.665 | 5.942 | 0.561 | 0.796 |
| Q03591 | Complement factor H-related protein 1 | -0.468 | 0.567 | 0.722 | 5.668 | 0.742 | 0.796 |
| P01034 | Cystatin C | 0.375 | 0.428 | 0.665 | -15.716 | 0.218 | 0.796 |
| P02679 | Fibrinogen gamma chain | -0.153 | 0.712 | 0.831 | 16.024 | 0.183 | 0.796 |
| Q15485 | Ficolin-2 | 0.364 | 0.383 | 0.665 | 9.066 | 0.387 | 0.796 |
| P01860 | Ig gamma-3 chain C region | 0.120 | 0.799 | 0.839 | 3.901 | 0.742 | 0.796 |
| P01834 | Ig kappa chain C region | -0.448 | 0.326 | 0.665 | -7.656 | 0.515 | 0.796 |
| P05362 | Intercellular adhesion molecule 1 | 0.463 | 0.228 | 0.665 | 10.823 | 0.328 | 0.796 |
| P36955 | Pigment epithelium-derived factor | 1.866 | 0.294 | 0.665 | 18.559 | 0.390 | 0.796 |
| P13501 | RANTES | 0.860 | 0.139 | 0.665 | 5.353 | 0.659 | 0.796 |
| P02787 | Serotransferrin | -0.770 | 0.384 | 0.665 | -8.979 | 0.449 | 0.796 |
| P02766 | Transthyretin | -0.402 | 0.421 | 0.665 | -4.532 | 0.717 | 0.796 |

**Supplementary Table 18. Associations of proteins with CSF pTau in AD in the EMIF study.** Abbreviations: Uniprot, universal protein resource. Nominally significant **P* < 0.05.

| **UniProt ID** | **Protein Name** | **Logistic Regression** | | | **Linear Regression** | | |
| --- | --- | --- | --- | --- | --- | --- | --- |
|  |  | **β** | ***P* value** | ***q* value** | **β** | ***P* value** | ***q* value** |
| P02763 | Alpha-1-acid glycoprotein 1 | -0.447 | 0.235 | 0.992 | -7.267 | 0.155 | 0.544 |
| P01009 | Alpha-1-antitrypsin | 0.214 | 0.534 | 0.992 | 6.446 | 0.119 | 0.544 |
| P01023 | Alpha-2-macroglobulin | 0.167 | 0.674 | 0.992 | 1.429 | 0.817 | 0.895 |
| P02647 | Apolipoprotein A-I | -0.110 | 0.703 | 0.992 | -1.415 | 0.745 | 0.895 |
| P02656 | Apolipoprotein C-III | -0.033 | 0.947 | 0.992 | -10.250 | 0.150 | 0.544 |
| P55056 | Apolipoprotein C-IV | -0.163 | 0.601 | 0.992 | -7.881 | 0.096 | 0.544 |
| P08519 | Apolipoprotein(a) | 0.233 | 0.422 | 0.992 | 3.216 | 0.452 | 0.895 |
| P10909 | Clusterin | -0.003 | 0.992 | 0.992 | 1.271 | 0.798 | 0.895 |
| P0C0L5 | Complement C4-B | 0.040 | 0.904 | 0.992 | 0.990 | 0.825 | 0.895 |
| / | Complement component 4 | 0.217 | 0.437 | 0.992 | -4.475 | 0.282 | 0.845 |
| Q03591 | Complement factor H-related protein 1 | -0.114 | 0.786 | 0.992 | 9.229 | 0.140 | 0.544 |
| P01034 | Cystatin C | -0.153 | 0.635 | 0.992 | -3.134 | 0.525 | 0.895 |
| P02679 | Fibrinogen gamma chain | -0.075 | 0.762 | 0.992 | 1.277 | 0.750 | 0.895 |
| Q15485 | Ficolin-2 | -0.678 | 0.019* | 0.408 | -2.553 | 0.490 | 0.895 |
| P01860 | Ig gamma-3 chain C region | 0.050 | 0.852 | 0.992 | -2.470 | 0.517 | 0.895 |
| P01834 | Ig kappa chain C region | 0.259 | 0.363 | 0.992 | -1.258 | 0.761 | 0.895 |
| P05362 | Intercellular adhesion molecule 1 | 0.020 | 0.942 | 0.992 | -0.765 | 0.852 | 0.895 |
| P36955 | Pigment epithelium-derived factor | 0.226 | 0.690 | 0.992 | 1.002 | 0.897 | 0.897 |
| P13501 | RANTES | -0.327 | 0.181 | 0.992 | -2.424 | 0.565 | 0.895 |
| P02787 | Serotransferrin | 0.259 | 0.470 | 0.992 | 0.918 | 0.848 | 0.895 |
| P02766 | Transthyretin | -0.153 | 0.594 | 0.992 | -6.371 | 0.156 | 0.544 |

**Supplementary Table 19. Associations of proteins with CSF pTau in MCI in the EMIF study.** Abbreviations: Uniprot, universal protein resource. Nominally significant **P* < 0.05.

| **UniProt ID** | **Protein Name** | **Logistic Regression** | | | **Linear Regression** | | |
| --- | --- | --- | --- | --- | --- | --- | --- |
|  |  | **β** | ***P* value** | ***q* value** | **β** | ***P* value** | ***q* value** |
| P02763 | Alpha-1-acid glycoprotein 1 | -0.081 | 0.671 | 0.976 | -3.480 | 0.225 | 0.677 |
| P01009 | Alpha-1-antitrypsin | -0.030 | 0.849 | 0.976 | -2.565 | 0.261 | 0.677 |
| P01023 | Alpha-2-macroglobulin | 0.042 | 0.841 | 0.976 | -0.463 | 0.882 | 0.982 |
| P02647 | Apolipoprotein A-I | 0.191 | 0.279 | 0.877 | 1.609 | 0.524 | 0.823 |
| P02656 | Apolipoprotein C-III | -0.226 | 0.503 | 0.961 | -2.063 | 0.668 | 0.877 |
| P55056 | Apolipoprotein C-IV | 0.134 | 0.486 | 0.961 | 3.981 | 0.152 | 0.677 |
| P08519 | Apolipoprotein(a) | 0.032 | 0.849 | 0.976 | 1.919 | 0.420 | 0.801 |
| P10909 | Clusterin | 0.363 | 0.054 | 0.571 | 4.081 | 0.108 | 0.677 |
| P0C0L5 | Complement C4-B | 0.021 | 0.909 | 0.976 | 4.284 | 0.104 | 0.677 |
| / | Complement component 4 | -0.468 | 0.015* | 0.316 | -3.056 | 0.242 | 0.677 |
| Q03591 | Complement factor H-related protein 1 | -0.058 | 0.826 | 0.976 | -3.772 | 0.313 | 0.677 |
| P01034 | Cystatin C | -0.003 | 0.985 | 0.985 | -1.382 | 0.583 | 0.823 |
| P02679 | Fibrinogen gamma chain | -0.177 | 0.323 | 0.877 | -3.372 | 0.177 | 0.677 |
| Q15485 | Ficolin-2 | -0.026 | 0.869 | 0.976 | 3.808 | 0.096 | 0.677 |
| P01860 | Ig gamma-3 chain C region | 0.190 | 0.233 | 0.877 | 0.268 | 0.909 | 0.982 |
| P01834 | Ig kappa chain C region | -0.017 | 0.914 | 0.976 | -2.259 | 0.322 | 0.677 |
| P05362 | Intercellular adhesion molecule 1 | 0.156 | 0.334 | 0.877 | -0.033 | 0.989 | 0.989 |
| P36955 | Pigment epithelium-derived factor | 0.257 | 0.489 | 0.961 | 0.442 | 0.935 | 0.982 |
| P13501 | RANTES | -0.225 | 0.206 | 0.877 | 0.397 | 0.875 | 0.982 |
| P02787 | Serotransferrin | 0.213 | 0.254 | 0.877 | 1.906 | 0.468 | 0.819 |
| P02766 | Transthyretin | -0.017 | 0.929 | 0.976 | 1.533 | 0.588 | 0.823 |

**Supplementary Table 20. Associations of proteins with CSF pTau in CTL in the EMIF study.** Abbreviations: Uniprot, universal protein resource. Nominally significant **P* < 0.05, ***P* < 0.01.

| **UniProt ID** | **Protein Name** | **Logistic Regression** | | | **Linear Regression** | | |
| --- | --- | --- | --- | --- | --- | --- | --- |
|  |  | **β** | ***P* value** | ***q* value** | **β** | ***P* value** | ***q* value** |
| P02763 | Alpha-1-acid glycoprotein 1 | 0.859 | 0.578 | 1.000 | -1.650 | 0.380 | 0.792 |
| P01009 | Alpha-1-antitrypsin | -0.449 | 0.591 | 1.000 | 1.598 | 0.306 | 0.792 |
| P01023 | Alpha-2-macroglobulin | -0.481 | 0.742 | 1.000 | -0.467 | 0.854 | 0.993 |
| P02647 | Apolipoprotein A-I | -0.879 | 0.626 | 1.000 | -1.805 | 0.335 | 0.792 |
| P02656 | Apolipoprotein C-III | 3.045 | 0.321 | 1.000 | 2.242 | 0.504 | 0.819 |
| P55056 | Apolipoprotein C-IV | 1.045 | 0.398 | 1.000 | -0.005 | 0.998 | 0.998 |
| P08519 | Apolipoprotein(a) | 1.555 | 0.342 | 1.000 | 1.896 | 0.245 | 0.792 |
| P10909 | Clusterin | 1.864 | 0.283 | 1.000 | 3.165 | 0.099 | 0.694 |
| P0C0L5 | Complement C4-B | -75.749 | 0.999 | 1.000 | -0.599 | 0.744 | 0.976 |
| / | Complement component 4 | 0.846 | 1.000 | 1.000 | 0.854 | 0.534 | 0.819 |
| Q03591 | Complement factor H-related protein 1 | 0.323 | 0.772 | 1.000 | 1.128 | 0.652 | 0.913 |
| P01034 | Cystatin C | -5.326 | 0.250 | 1.000 | -1.511 | 0.415 | 0.792 |
| P02679 | Fibrinogen gamma chain | 0.533 | 0.609 | 1.000 | 3.342 | 0.054 | 0.694 |
| Q15485 | Ficolin-2 | -0.253 | 0.752 | 1.000 | -0.103 | 0.946 | 0.993 |
| P01860 | Ig gamma-3 chain C region | 1.240 | 0.198 | 1.000 | 0.135 | 0.937 | 0.993 |
| P01834 | Ig kappa chain C region | -2.401 | 0.147 | 1.000 | -1.929 | 0.252 | 0.792 |
| P05362 | Intercellular adhesion molecule 1 | 34.256 | 0.999 | 1.000 | 2.111 | 0.186 | 0.792 |
| P36955 | Pigment epithelium-derived factor | -48.859 | 1.000 | 1.000 | 1.751 | 0.546 | 0.819 |
| P13501 | RANTES | -4553.463 | 0.971 | 1.000 | -1.524 | 0.385 | 0.792 |
| P02787 | Serotransferrin | -20.752 | 1.000 | 1.000 | -2.679 | 0.095 | 0.694 |
| P02766 | Transthyretin | -0.123 | 0.885 | 1.000 | -0.453 | 0.803 | 0.992 |
