# Supplementary material for: Plasma Protein Biomarkers for the Prediction of CSF Amyloid and Tau and [18F]-Flutemetamol PET Scan Result
Source: Front Aging Neurosci. 2018 Dec 11;10:409. doi: 10.3389/fnagi.2018.00409 (PMC6297196; doi:10.3389/fnagi.2018.00409)
Supplement: Supplementary file 3 [file Data_Sheet_3.docx]

**Supplementary Table 21. IEC and IRB list for the GE-067-005 cohort.**

| Center Number | IEC/IRB Affiliation | Chairman |
| --- | --- | --- |
| 001 | John Hopkins University School of Medicine Institutional  Review Board  1620 McElderry Street  Reed Hall, Room 130  Baltimore, MD 21205  USA | David Cornblath, MD |
| 002 | Medical School Institutional Review Board  Argus I Building  517 W. William  Ann Arbor, MI 48103-4943  USA | Michael Geisser  John Weg |
| 003 | University of Pittsburgh Institutional Review Board  3500 Fifth Avenue  Hieber Building, Ground Floor  Pittsburgh, PA 15213  USA | Ronald Shapiro, MD |
| 004 | Mayo Institutional Review Board  4-60, 201 Bldg  Mayo Clinic  200 1st St. SW  Rochester, MN 55905  USA | Joseph Lobl, MD |
| 005 | University of Pennsylvania Office of Regulatory Affairs  3624 Market Street, Suite 301 S  Philadelphia, PA 19104  USA | Dr. Emma Meagher |
| 006, 007, 016 | Medical Ethics Committee UZ KU Leuven/Clinical  Research  Gasthuisberg University Hospital E330  Herestraat 49  3000 Leuven  Belgium | Prof W Van den Bogaert |
| 008 | Commission d'éthique bio-médicale hospitalo-facultaire  Avenue Hipprocrate 55/14 - Tour Harvey  1200 Bruxelles  Belgium | Prof J-M Maloteaux |
| 009 | Comité d'éthique hospito-facultaire de l'Université de Liège  Centre hospitalier universitaire  Domaine du Sart Tilman  4000 Liège  Belgium | Prof V Seutin |
| 010, 017, 034 | De Videnskabsetiske komiteer i region Hovedstaden  Regionsgården  Kongens Vænge 2  DK 3400 Hillerød  Denmark |  |
| 012 | Regionala Etikprövningsnämnden i Lund  Box 133  221 00 Lund  Sweden | Jacob Branting,  Administrative Secretary |
| 013 | STM/ETENE, TUKIJA  POB 33  00023 Valtioneuvosto  Finland | Outi Konttinen |
| 014, 015, 018, 019, 020, 022, 023, 024, 028, 037 | Hammersmith, Queen Charlotte's & Chelsea Research  Ethics Committee  Room 4W/12, 4th Floor  Charing Cross Hospital  Fulham Palace Road  London  W6 8RF  UK | Mr Clive Collett |
| 021 | Michigan State University, Biomedical and Health  Institutional Review Board  207 Olds Hall  East Lansing, MI 48824  USA | Ashir Kumar, MD |
| 025 | Comité d'Ethique  Hospital Erasme  808 Route de Lennik  1070 Brussels  Belgium | Prof A Herchuels |
| 026 | Ethisch Comité  Universitair Ziekenhuis Antwerpen  Wilrijkstraat 10  2650 Edegem  Belgium | Prof Patrick Cras |
| 027, 029, 030, 032, 035, 036 | Western Institutional Review Board  3535 Seventh Avenue, SW  Olympia, WA 98502  USA | Theodore D. Schultz |
| 031 | Mount Sinai Medical Center  4300 Alton Road, Pearlman Bldg  Miami Beach, FL 33140  USA | Jose A. Adams, MD |
| 033 | Catholic Healthcare West/St. Joseph's Hospital & Medical  Center  350 W. Thomas Road  Phoenix, AZ 85013  USA | C. Phillip Daspit, MD |

| **Supplementary table 22. Ethical approval committee of each center for the EMIF 500 cohort.** | | | |
| --- | --- | --- | --- |
| **Center** | **Part of multicenter** | **Country** | **Approval Committee** |
| Aristotle University, Thessaloniki | DESCRIPA, EDAR, Pharmacog | Greece | Aristotle University of Thessaloniki Medical School Ethics Committee |
| Central Institute for Mental Health, Mannheim | EDAR | Germany | Ethics Committee of the Medical Faculty Mannheim, University of Heidelberg |
| GAP, San Sebastian | - | Spain | Ethic and Clinical Research Committee Donostia |
| Hôpital Timone Adultes, Marseille | Pharmacog | France | Ethics committee Inserm and Aix Marseille University |
| Hospital Clínic de Barcelona IDIBAPS | Pharmacog | Spain | The Healthcare Ethics Committee of the Hospital Clínic |
| Hospital de la Santa Creu i Sant Pau, Barcelona | EDAR | Spain | Central Clinical Research and Clinical Trials Unit (UICEC Sant Pau) |
| INSERM, Toulouse | Pharmacog | France | INSERM Ethical Committee |
| IRCCS-FBF, Brescia | Pharmacog | Italy | Ethic Committee of the IRCCS San Giovanni di Dio FBF |
| IRCCS-SDN, Napels | Pharmacog | Italy | Comitato Etico IRCCS Pascale - Napoli |
| Karolinska Institutet, Stockholm | EDAR | Sweden | Ethics Committee at Karolinska Institutet |
| Katholieke Universiteit, Leuven | EDAR | Belgium | Ethische commissie onderzoek UZ/KU Leuven |
| Lausanne University Hospital, Lausanne | - | Switzerland | Research Ethics Committee Lausanne University Hospital |
| Maastricht University, Maastricht | DESCRIPA, EDAR | Netherlands | Medical ethical committee Maastricht University Medical Center |
| Rigshospitalet, Copenhagen | EDAR | Denmark | Committee on Health Research Ethics, Region of Denmark |
| University of Mediterranean, Marseille | Pharmacog | France | Ethics committee of Mediterranean University |
| University of Lille, Lille | Pharmacog | France | University of Lille Ethics committee |
| University of Leipzig, Leipzig | Pharmacog | Germany | Ethical Committee at the Medical Faculty, Leipzig University |
| University of Essen, Essen | Pharmacog | Germany | Ethical Committee at the Medical Faculty, University Hospital Essen |
| University of Antwerp, Antwerp | - | Belgium | Ethics committee University of Antwerp |
| University of Genoa, Genoa | Pharmacog | Italy | Ethical Committee of University of Genoa |
| University of Gothenburg, Gothenburg | - | Sweden | Ethics Committee, University of Gothenburg |
| University of Perugia, Perugia | Pharmacog | Italy | Human ethics Committee of the University of Perugia |
| VU Medical Center, Amsterdam | EDAR, Pharmacog | Netherlands | Medical ethics committee VU Medical Center |
