# Supplementary material for: Plasma Protein Biomarkers for the Prediction of CSF Amyloid and Tau and [18F]-Flutemetamol PET Scan Result
Source: Front Aging Neurosci. 2018 Dec 11;10:409. doi: 10.3389/fnagi.2018.00409 (PMC6297196; doi:10.3389/fnagi.2018.00409)
Supplement: Supplementary file 4 [file Data_Sheet_4.docx]

**1 Supplementary methods. Blood sample collection and analysis in GE067-005 study cohort**

For collection of plasma samples, pre-chilled 10 mL EDTA tubes were filled completely with blood and mixed immediately by gently inverting 8-10 times. Within 15 minutes of collection, the tubes were centrifuged for 10 min at 1500g in a refrigerated centrifuge. The plasma was transferred into pre-chilled cryovials and frozen immediately at -70°C until ready to use.
